# Early Production of IL-22 but Not IL-17 by Peripheral Blood Mononuclear Cells Exposed to live *Borrelia burgdorferi*: The Role of Monocytes and Interleukin-1

**DOI:** 10.1371/journal.ppat.1001144

**Published:** 2010-10-14

**Authors:** Malte Bachmann, Katharina Horn, Ina Rudloff, Itamar Goren, Martin Holdener, Urs Christen, Nicole Darsow, Klaus-Peter Hunfeld, Ulrike Koehl, Peter Kind, Josef Pfeilschifter, Peter Kraiczy, Heiko Mühl

**Affiliations:** 1 *pharmazentrum frankfurt*/ZAFES, University Hospital Goethe University Frankfurt, Frankfurt, Germany; 2 Institute of Medical Microbiology and Infection Control, University Hospital Goethe University Frankfurt, Frankfurt, Germany; 3 Institute for Laboratory Medicine, North West Medical Centre, Frankfurt, Germany; 4 Pediatric Hematology and Oncology, University Hospital Goethe-University Frankfurt, Frankfurt, Germany; 5 Dermatohistological Laboratory Offenbach, Offenbach, Germany; Medical College of Wisconsin, United States of America

## Abstract

If insufficiently treated, Lyme borreliosis can evolve into an inflammatory disorder affecting skin, joints, and the CNS. Early innate immunity may determine host responses targeting infection. Thus, we sought to characterize the immediate cytokine storm associated with exposure of PBMC to moderate levels of live *Borrelia burgdorferi*. Since Th17 cytokines are connected to host defense against extracellular bacteria, we focused on interleukin (IL)-17 and IL-22. Here, we report that, despite induction of inflammatory cytokines including IL-23, IL-17 remained barely detectable in response to *B. burgdorferi*. In contrast, T cell-dependent expression of IL-22 became evident within 10 h of exposure to the spirochetes. This dichotomy was unrelated to interferon-γ but to a large part dependent on caspase-1 and IL-1 bioactivity derived from monocytes. In fact, IL-1β as a single stimulus induced IL-22 but not IL-17. Neutrophils display antibacterial activity against *B. burgdorferi*, particularly when opsonized by antibodies. Since neutrophilic inflammation, indicative of IL-17 bioactivity, is scarcely observed in *Erythema migrans*, a manifestation of skin inflammation after infection, protective and antibacterial properties of IL-22 may close this gap and serve essential functions in the initial phase of spirochete infection.

## Author Summary

Lyme borreliosis displays multifaceted clinical manifestations caused by the *Borrelia burgdorferi sensu lato complex*. If insufficiently treated, infection may proceed to inflammatory complications of chronic infection. Th17-like cytokines, foremost IL-17 and IL-22, are crucial for host defense against extracellular bacteria. IL-17/IL-22 secretion by human leukocytes exposed to live *Borreliae* has not been analyzed. Here we report that *B. burgdorferi*-activated PBMC lack immediate IL-17 expression despite being highly activated and robust T cell-dependent production of IL-22 that to a large part is mediated by monocyte-derived IL-1. Early innate immunity may shape dermal infection, thus likely affecting bacterial dissemination. Specifically, insufficient neutrophil recruitment/function, supposedly due to insufficient early IL-17 production along with a lack of opsonizing antibodies, may favor the spread of *B. burgdorferi*. Indeed, neutrophilic inflammation, indicative of IL-17 bioactivity, is scarcely observed in *Erythema migrans*, a manifestation of skin inflammation after infection. Production of IL-22 may fill this gap. Current knowledge on the role of IL-22 in epithelial biology in fact supports the hypothesis that IL-22 may serve as protection, particularly under conditions of inadequate neutrophil-driven host defense as seen early in *B. burgdorferi* infection.

## Introduction

Interleukin (IL)-22, along with IL-17, is principally recognized as decisive component of Th17-like immune responses in humans and mice [1]–[7]. Beyond that, Th1 and distinct NK cell subsets have been identified as relevant sources of IL-22 [1], [2], [8]. Due to restricted expression of IL-22RI, this cytokine characteristically affects almost exclusively the non-leukocytic cell compartment, in particular hepatocytes, synoviocytes, and cells of epithelial origin such as keratinocytes and colonocytes. Being a member of the IL-10 cytokine family, activation of the signal transducer and activator of transcription (STAT) pathway, foremost STAT3, obviously plays a pivotal role for IL-22 immunomodulatory and tissue-protective properties [1], [2]. Enhanced IL-22 levels have been linked to various states of immunoactivation as seen in the context of infection [9]–[12], autoimmunity [13], [14], and allergic disorders [6]. However, the role of IL-22 in disease is not unambiguous but apparently depends on the pathophysiological context. Specifically, IL-22 ameliorated disease in selected models of microbe/infection-driven inflammation at host/environment interfaces [9], [10], [15], [16]. This property likely relates to upregulation of anti-microbial proteins such as β-defensins, regIII proteins and lipocalin-2 [1], [9], [10], of anti-bacterial inducible nitric oxide synthase (iNOS) [17], and to enhanced mucus production under the influence of IL-22 [15]. In contrast, data in the context of psoriasis [18], [19] and arthritis [13] suggest a pathogenic function of this cytokine. Notably, those latter inflammatory diseases are not primarily infection-driven but linked to autoimmunity and tissue hyperplasia.

Recent research efforts aiming to further understand the function of specific T cell subsets in shaping immune responses revealed considerable plasticity and species specificity concerning the development and fate of Th17 cells and their profile of cytokine production. Not only has now been widely appreciated that a considerable proportion of IL-17^+^ Th17 cells also expresses the Th1 signature cytokine interferon (IFN)-γ [4], [6], [20]–[22]. Moreover, IL-22^+^ IL-17^-^ T cells that do not fit the Th1/Th2/Th17 classification were recently introduced. These T cells have lately been coined Th22 or T22, though further characterization of those recent subsets appears crucial [23]–[27].

Lyme borreliosis, the most common vector-borne disease in the United States and Europe, is characterized by multifaceted clinical manifestations caused by spirochetes of the *Borrelia burgdorferi sensu lato complex*, in particular *B. afzelii, B. garinii, B. spielmanii, B. bavariensis*, and *B. burgdorferi*. If left insufficiently- or untreated, infection may proceed to cutaneous manifestations, carditis, neuroborreliosis, or Lyme arthritis, representing an array of most relevant and severe inflammatory complications of chronic infection [28], [29]. The pattern of early cytokine production not only may determine initial flu-like symptoms as seen in a number of these patients but also dissemination and hence course of disease in Lyme borreliosis [30], [31]. By using human peripheral blood mononuclear cells (PBMC), a culture model of reasonably manipulated primary leukocytic cells, we set out to characterize herein the cytokine storm initiated by exposure to moderate levels of *B. burgdorferi* for up to 65h. Since Th17-like immune responses have been connected in particular to host defense against extracellular bacteria [4], we chose to focus on expression of IL-17 and IL-22.

## Results

### A specific cytokine pattern associated with PBMC exposed to live *B. burgdorferi*

**Figure 1:**
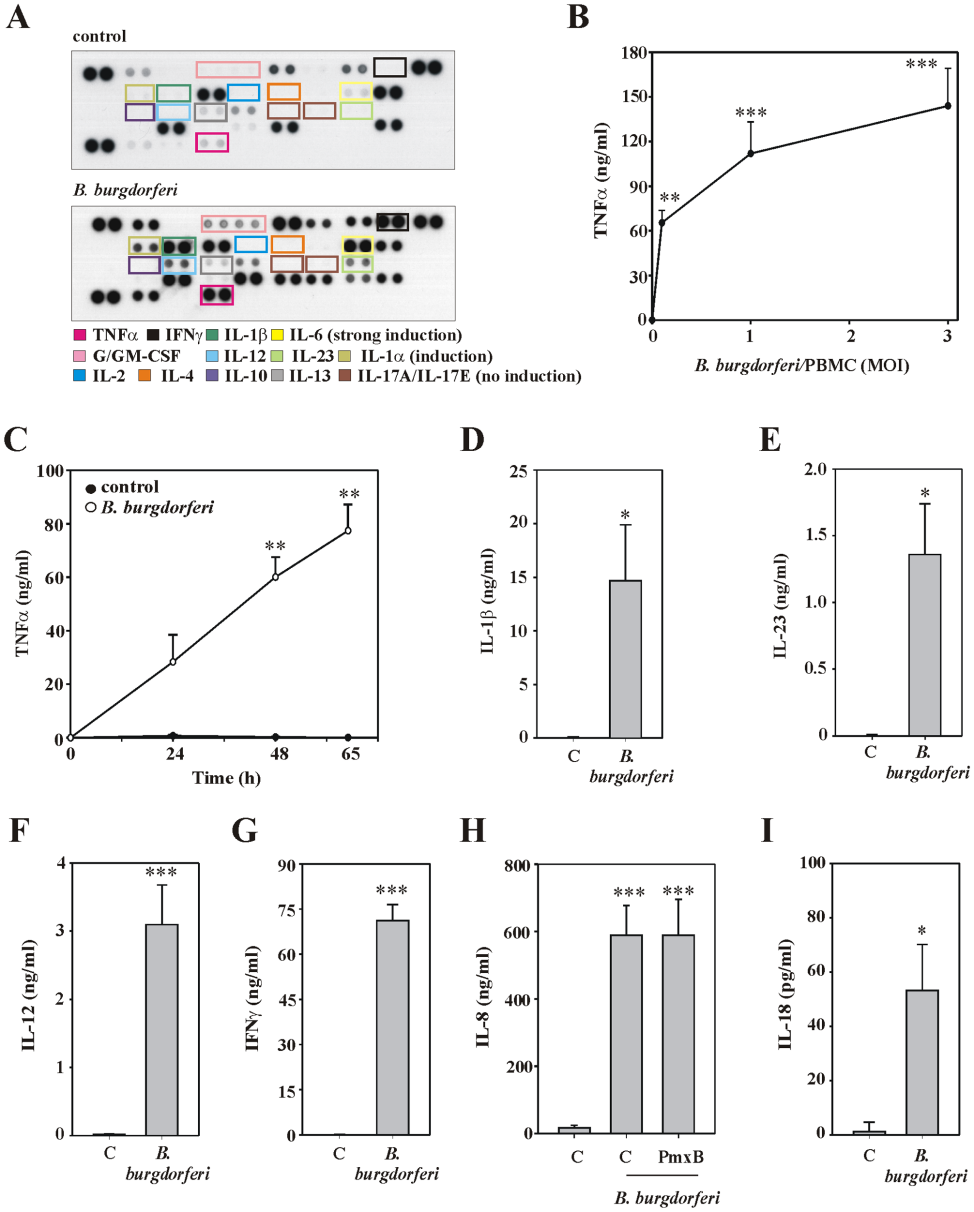
A specific cytokine pattern associated with PBMC exposed to live *B. burgdorferi.* (A) PBMC were either kept as unstimulated control or exposed to *B. burgdorferi* 297 (MOI = 0.1). After 65 h cell-free cell culture supernatants were analyzed in a 1∶3 dilution by antibody array analysis. One representative of two independently performed experiments (two different donors) is shown. (B) PBMC were either kept as unstimulated control or exposed to the indicated MOI of *B. burgdorferi* 297. After 65 h, TNFα release was determined by ELISA. Data are expressed as means ± SEM (n = 6 for unstimulated control and MOI = 0.1, n = 3 for MOI = 1 and 3); **p<0.01, ***p<0.001 compared with unstimulated control; raw data were analyzed by one-way ANOVA with post hoc Bonferroni correction. (C) PBMC were either kept as unstimulated control or were exposed to *B. burgdorferi* 297 (MOI = 0.1). After the indicated time periods TNFα release was determined by ELISA. Data are expressed as means ± SEM (n = 3); **p<0.01, compared with unstimulated control at the respective time point; raw data were analyzed by unpaired Student's *t*-test. (D-I) PBMC were either kept as unstimulated control or were exposed to *B. burgdorferi* 297 (MOI = 0.1). After 24 h (D-G, I) or 65 h (H), release of IL-1β (D; n = 3; *p<0.05, compared with unstimulated control), IL-23 (E; n = 3; *p<0.05, compared with unstimulated control), IL-12 (F; n = 5; ***p<0.001, compared with unstimulated control), IFNγ (G; n = 3; ***p<0.001, compared with unstimulated control), IL-8 (H; n = 5; ***p<0.001, compared with unstimulated control; where indicated experiments were performed in the presence of PmxB at 3 µg/ml), and IL-18 (I; n = 3; *p<0.05, compared with unstimulated control) was determined by ELISA. Raw data were either analyzed by unpaired Student's *t*-test (D-G, I) or by one-way ANOVA with post hoc Bonferroni correction (H).

Here, we sought to investigate in detail initial cytokine production by PBMC in response to live *B. burgdorferi*. Protein profiling by antibody array analysis indicated a distinct pattern of cytokine secretion by PBMC under the influence of live spirochetes. Specifically, we confirm previous observations on activation of prototypic pro-inflammatory cytokines such as IL-1β, tumor necrosis factor (TNF)-α, IL-6, and IFNγ [32]–[34]. Moreover, we report for the first time on secretion of IL-12 and IL-23 from PBMC activated by live *B. burgdorferi* (**Fig. 1A**). An array overview and a semiquantitative analysis of cytokine profiling shown in Figure 1A is provided in supplementary data (**Fig. S1 and S2**). Array data on *B. burgdorferi*-induced secretion of TNFα (**Fig. 1BC**), IL-1β (**Fig. 1D**), IL-23 (**Fig. 1E**), IL-12 (**Fig. 1F**), and IFNγ (**Fig. 1G**) were confirmed by independent sets of experiments and ELISA analysis. Unless otherwise indicated, in the current study we chose to expose PBMC to highly viable and motile *B. burgdorferi* cells at a MOI of 0.1. This low 1/10 (*B. burgdorferi*/PBMC) ratio was specifically selected because at this MOI the spirochete is not supposed to induce cell death in either monocytes or T cells [33]. This also allows prolonged incubation periods for analysis of cytokines that are induced more slowly or are dependent on activation of intermediate cell types.

IL-8 upregulation did not show up in array analysis which was due to common background expression of IL-8 in freshly-isolated PBMC [35] and analysis under saturating assay conditions. Yet, ELISA analysis (**Fig. 1H**) confirmed IL-8 induction by *B. burgdorferi* [34], [36], [37] which was not affected by coincubation with polymyxin B (PmxB). The latter observation agrees with absence of LPS in *Borreliae* and clearly excludes LPS as stimulatory component in the cellular model used herein. Array analysis also confirmed previous data on basal production of IL-1Ra [38], macrophage migration inhibitory factor (MIF) [39], and interferon-inducible protein-10 (IP-10) [40] by PBMC which over time (65 h incubation period) obviously precluded (semi)-quantitative evaluation of those three parameters under the current assay conditions (**Fig. S2**).

Most notably, array analysis also identified a set of cytokines that (at a MOI of 0.1) remained at undetectable levels despite strong activation of PBMC. This group of cytokines included IL-2, IL-4, IL-10, IL-13, and most remarkably IL-17A (denoted as IL-17 throughout this manuscript) as well as IL-17E (**Fig. 1A**). Lack of upregulation of IL-2 (5.9 pg/ml±1.5 pg/ml versus 2.6 pg/ml±1.3 pg/ml for control versus *B. burgdorferi* 297, MOI = 30, 65 h incubation, n = 5) and IL-17 (7.1 pg/ml±2.2 pg/ml versus 8.8 pg/ml±3.0 pg/ml for control versus *B. burgdorferi* 297, MOI = 30, 65 h incubation, n = 5) was unmistakably confirmed by ELISA analysis of additional independent experiments with a MOI of up to 30. In addition, secretion of IL-18 was also assessed by ELISA. **Fig. 1I** demonstrates only modest IL-18 secretion in response to *B. burgdorferi*, an observation in striking contrast to stunning secretion of IL-1β in those same experiments (**Fig. 1D**).

### Early production of IL-22 but not of IL-17 by PBMC exposed to live *B. burgdorferi*

**Figure 2:**
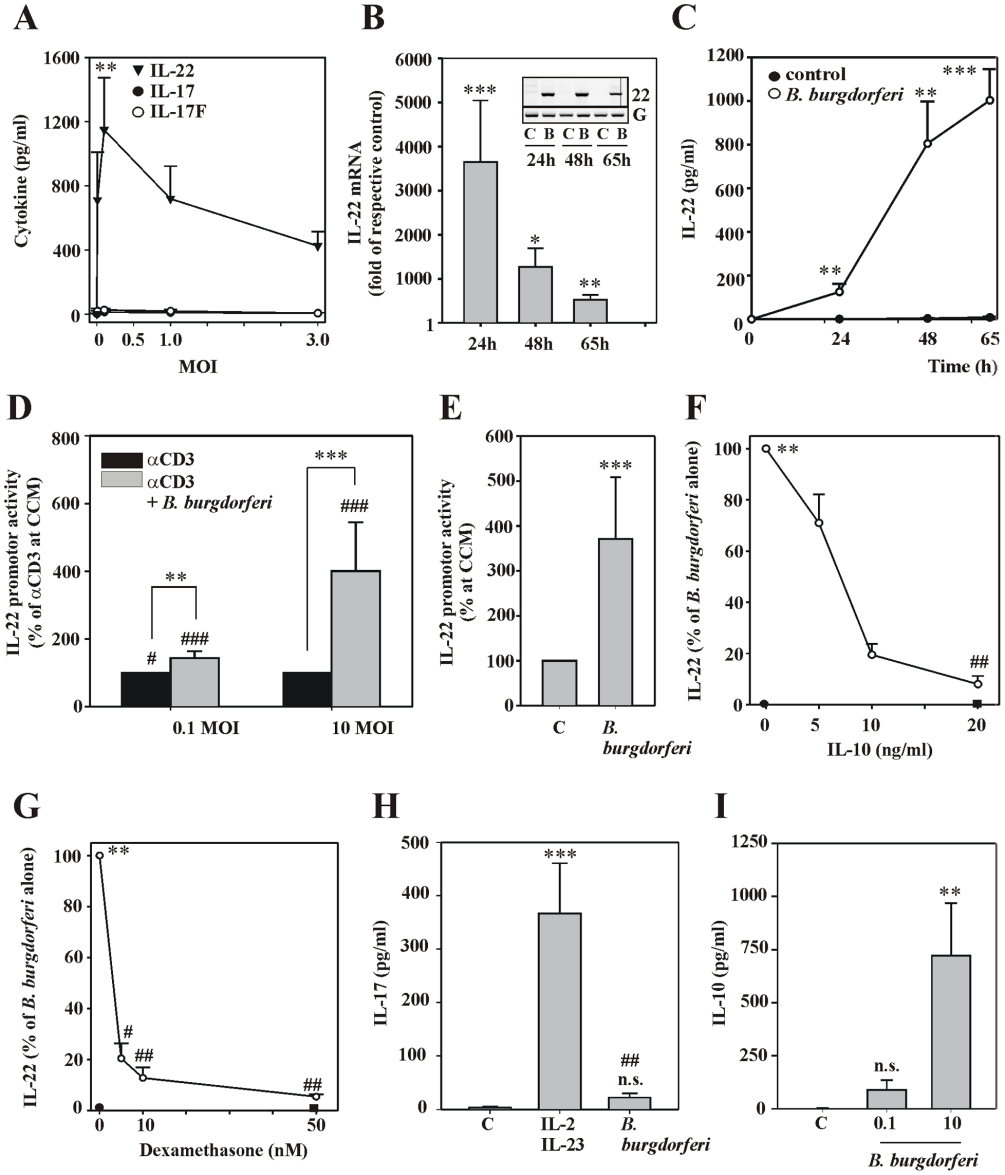
Production of IL-22 but not of IL-17 by PBMC exposed to live *B. burgdorferi*. (A) PBMC were either kept as unstimulated control or exposed to the indicated MOI of *B. burgdorferi* 297. After 65 h, IL-22 (closed triangles), IL-17 (closed circles), and IL-17F (open circles) release was determined by ELISA. Data are expressed as means ± SEM (n = 10); **p<0.01, compared with unstimulated control; raw data were analyzed by one-way ANOVA with post hoc Bonferroni correction. (B) PBMC were either kept as unstimulated control or were exposed to *B. burgdorferi* 297 (MOI = 0.1). After the indicated incubation periods, total RNA was isolated and IL-22 mRNA expression was determined by real-time PCR analysis. IL-22 mRNA was normalized to that of GAPDH and is shown as mean fold induction compared to unstimulated control (at the respective time point) ± SEM (n = 3); *p<0.05, **p<0.01, ***p<0.001 compared with unstimulated control (at the respective time point); raw data were analyzed by unpaired Student's *t*-test. Inset: In addition to real-time PCR, data from one representative experiment was analyzed by standard PCR analysis. (C) PBMC were either kept as unstimulated control or were exposed to *B. burgdorferi* 297 (MOI = 0.1). After the indicated time periods IL-22 release was determined by ELISA. Data are expressed as means ± SEM (n = 5); **p<0.01, ***p<0.001 compared with unstimulated control at the respective time point; raw data were analyzed by unpaired Student's *t*-test. (D) Jurkat T cells were transfected for 5 h with pGL3-IL22 together with *Renilla* luciferase as described in the *materials and methods* section. After 15 h of rest, cells were resuspended in control conditioned media (CCM) obtained from unstimulated PBMC or in conditioned media from PBMC exposed to *B. burgdorferi* 297 (either MOI = 0.1 (n = 3) or MOI = 10 (n = 6), stimulation period: 15 h) and stimulated with αCD3 (25 µg/ml) thereafter. After another 8 h, cells were harvested and luciferase assays were performed. Data are expressed as promoter activity (% of αCD3 at CCM ± SD); **p<0.01, ***p<0.001 compared with αCD3 at CCM; ^#^p<0.05, ^###^p<0.001 compared with Jurkat T cells in CCM in the absence of αCD3 and *B. burgdorferi*. Raw data were analyzed by one-way ANOVA with post hoc Bonferroni correction. (E) Jurkat T cells were treated using the protocol described under (D). However, only *B. burgdorferi* at a MOI of 10 were used to generate PBMC-derived conditioned media. In addition, αCD3 stimulation of Jurkat T cells was omitted. Data are expressed as promoter activity (% of CCM ± SD) (n = 8); ***p<0.001 compared with promoter activity at CCM. Raw data were analyzed by unpaired Student's *t*-test. (FG) PBMC were exposed to *B. burgdorferi* 297 (MOI = 0.1) either alone or in combination with the indicated concentrations of IL-10 (F) or dexamethasone (G). After 65 h, IL-22 release was determined by ELISA. Data (% of *B. burgdorferi* alone) are expressed as means ± SEM (IL-10: n = 6, dexamethasone: n = 3); closed circles denote unstimulated control, closed squares denote IL-10 at 20 ng/ml (F) or dexamethasone at 50 nM (G) alone; **p<0.01 compared with unstimulated control; ^#^p<0.05, ^##^p<0.01 compared with PBMC exposed to *B. burgdorferi* 297 alone; raw data were analyzed by one-way ANOVA with post hoc Bonferroni correction. (H) PBMC were either kept as unstimulated control or stimulated with IL-2 (20 ng/ml)/IL-23 (20 ng/ml) or were exposed to *B. burgdorferi* 297 (MOI = 0.1). After 65 h, IL-17 release was determined by ELISA. Data are expressed as means ± SEM (n = 15 for control and IL-2/IL-23, n = 8 for *B. burgdorferi* 297); n.s. denotes not significantly different from unstimulated control; ***p<0.001, compared with unstimulated control, ^##^p<0.01 compared with IL-2/IL-23; raw data were analyzed by one-way ANOVA with post hoc Bonferroni correction. (I) PBMC were either kept as unstimulated control or were exposed to the indicated dosages of *B. burgdorferi* 297. After 42 h, IL-10 release was determined by ELISA. Data are expressed as means ± SEM (n = 8 for control and *B. burgdorferi* 297 at a MOI of 0.1, n = 6 for *B. burgdorferi* 297 at a MOI of 10); n.s. denotes not significantly different from unstimulated control; **p<0.01, compared with unstimulated control; raw data were analyzed by one-way ANOVA with post hoc Bonferroni correction.

Array analysis revealed lack of immediate IL-17 production by PBMC in response to live *B. burgdorferi*. Hence, production of IL-22, a cytokine not covered by the antibody array used herein but usually produced in conjunction with IL-17 in Th17-like responses, was assessed under these same conditions. Surprisingly, ELISA analysis proved robust IL-22 induction by *B. burgdorferi* 297. Detailed analysis suggested optimal IL-22 secretion at the low MOI of 0.1. No induction of either IL-17 or IL-17F was detectable in these same experiments (**Fig. 2A**). Time-course analysis furthermore revealed strong and immediate induction of IL-22 mRNA that reached maximal levels within the first 24 h of exposure to *B. burgdorferi* 297 as detected by real-time PCR (**Fig. 2B**) and standard PCR analysis (**Fig. 2B, inset**), respectively. In fact, significant expression of IL-22 was detectable by real-time PCR as early as 10 h after onset of exposure of cells to B. burgdorferi 297 at a MOI of 0.1 (397.5-fold induction compared to unstimulated control, n = 3, p<0.05). IL-22 mRNA translated well into protein release that was most pronounced between 24 h–48 h of stimulation (**Fig. 2C**). The latter data agree with steady TNFα secretion over time (**Fig. 1C**) and altogether concure with lack of *B. burgdorferi*-mediated cytotoxicity at the low MOI of 0.1. No induction of IL-17 was detectable in these same time-course experiments at any data point of the 65 h incubation period. Levels of IL-17 always remained below 40 pg/ml in unstimulated control cells and in cells under the influence of *B. burgdorferi* 297 (data not shown). Since levels of IL-22 mRNA in unstimulated PBMC are extremely low, their upregulation in response to *Borreliae* was likely driven by gene promoter activation. To test this hypothesis a 1.230 bp human IL-22 promoter fragment was cloned into pGL3 and used for analysis of promoter activation by luciferase reporter assays. For that purpose conditioned media of either unstimulated (control conditioned medium, CCM) or *B. burgdorferi*-stimulated PBMC were generated and analyzed for their capability to amplify IL-22 promoter activity in the cellular context of αCD3-stimulated Jurkat T cells. Luciferase reporter assays shown in **Fig. 2D** demonstrate that conditioned media from PBMC exposed to spirochetes were in fact able to dose-dependently enhance IL-22 promoter activity. Moreover, conditioned media derived from PBMC exposed to *B. burgdorferi* at the high MOI of 10 significantly induced IL-22 promoter activity even in the absence of αCD3 stimulation (**Fig. 2E**). Strong inhibition of IL-22 production by coincubation of PBMC with IL-10 (**Fig. 2F**) or dexamethasone (**Fig. 2G**), both agents known for their pronounced monocyte deactivation potential, suggests that IL-22 secretion is largely dependent on prototypic pro-inflammatory signaling pathways. Induction of IL-22 was not restricted to *B. burgdorferi* 297 but was also seen in response to other spirochetes of the *B. burgdorferi sensu lato complex*. In contrast, all spirochetes tested were unable to mediate production of IL-17 (**Tab. 1**). Lack of IL-17 secretion by *B. burgdorferi*-activated PBMC was not related to a general incapacity of cultivated PBMC to rapidly produce IL-17. In fact, we confirm a previous report [22] on the ability of IL-2 to upregulate IL-17 release by PBMC (data not shown). Accordingly, the combination IL-2/IL-23 mediated significant secretion of IL-17 (**Fig. 2H**). Lack of IL-17 secretion by PBMC as detected herein was furthermore unrelated to a potential regulatory effect of IFNγ on immunoactivation and Th17-like cytokine responses [5], [41], [42]. In fact, coincubation with an IFNγ neutralizing antibody did not install IL-17 release by *B. burgdorferi*-stimulated PBMC (data not shown). In contrast to IL-17, production of IL-10 increased with increasing dosage of *B. burgdorferi* (**Fig. 2I**), which agrees with previous observations [34]. In fact, induction of IL-10 may relate to the tendency of reduced release of IL-22 (**Fig. 2A**) and TNFα (data not shown) after exposure of PBMC to high concentrations of live *B. burgdorferi*.

**Table 1:** IL-22 and IL-17 production by PBMC exposed to different *Borrelia* species.

Strain	IL-22 (pg/ml)	IL-17 (pg/ml) n = 4
Control	31.6**±**19.5 (n = 10)	5.7**±**4.4
297 (*B. burgdorferi;* CSF isolate)	1605.0**±**703.9 * (n = 10)	11.1**±**6.4
A14S (*B. spielmanii;* skin isolate)	2329.4**±**954.6 * (n = 7)	16.8**±**9.9
G1 (*B. garinii;* CSF isolate)	821.7**±**277.5 * (n = 10)	6.6**±**4.0
MT-M8 (*B. lusitaniae*; tick isolate)	881.5**±**187.5 ** (n = 5)	0.4**±**0.4
PBi (*B. bavariensis*; CSF isolate)	1032.8**±**246.8 ** (n = 5)	0.3**±**0.3
FEM-1 D15 (*B. afzelii*; skin isolate, clonal)	890.9**±**167.7 *** (n = 5)	2.7**±**1.2

IL-22 and IL-17 production by PBMC exposed to different *Borrelia* species, *B. bavariensis* not yet validated (MOI = 0.1; 48 h incubation; data are expressed as means ± SEM;

*p<0.05,

**: p<0.01,

***: p<0.001 *versus* control; raw data were analyzed by Student's *t*-test with *Borrelia* exposure compared to the unstimulated control of the respective set of experiments).

### IL-1 drives production of IL-22 by PBMC

**Figure 3:**
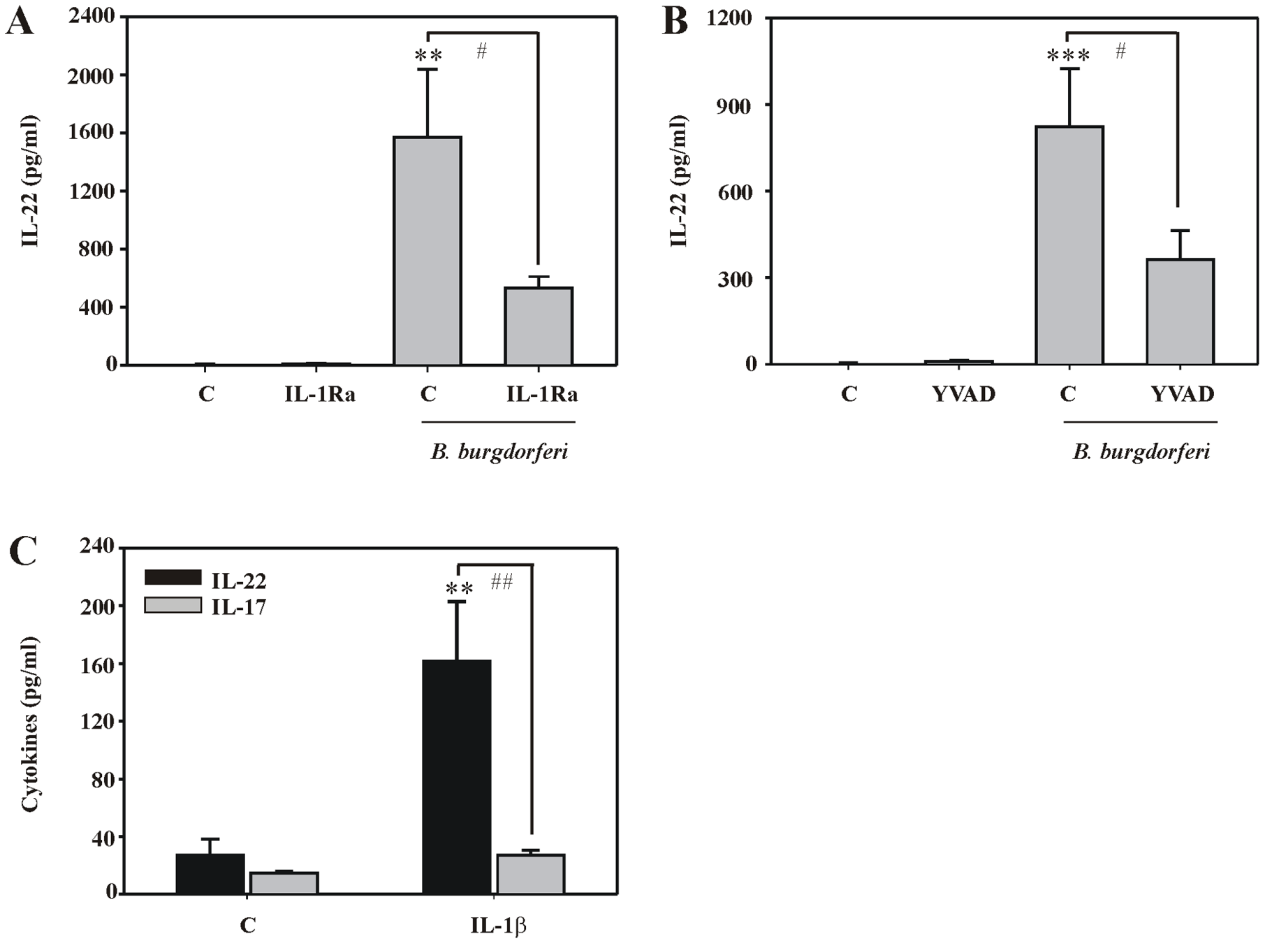
IL-1 drives production of IL-22 by PBMC exposed to live *B. burgdorferi*. (A) PBMC were kept as unstimulated control or cultivated together with IL-1Ra (1.3 µg/ml) in the presence or absence of *B. burgdorferi* 297 (MOI = 0.1). After 65 h, IL-22 release was determined by ELISA. Data are expressed as means ± SEM (n = 5); **p<0.01 compared with unstimulated control, ^#^p<0.05 compared with *B. burgdorferi* 297/IL-1Ra. (B) PBMC were kept as unstimulated control or cultivated together with Ac-YVAD-CHO (50 µM) in the presence or absence of *B. burgdorferi* 297 (MOI = 0.1). After 48 h, IL-22 release was determined by ELISA. Data are expressed as means ± SEM (n = 12); ***p<0.001 compared with unstimulated control, ^#^p<0.05 compared with *B. burgdorferi* 297/Ac-YVAD-CHO. (AB) IL-1Ra and Ac-YVAD-CHO were added to the cultures 0.5 h before exposure of PBMC to *B. burgdorferi*. (C) PBMC were either kept as unstimulated control or stimulated with IL-1β (50 ng/ml). After 65 h, IL-22 (black bars) and IL-17 (grey bars) release was determined by ELISA. Data are expressed as means ± SEM (n = 7); **p<0.01, compared with unstimulated control, ^##^p<0.01 compared with IL-17 levels in the presence of IL-1β. (ABC) Raw data were analyzed by one-way ANOVA with post hoc Bonferroni correction.

Although a fully consistent picture on mechanisms mediating human Th17 development is lacking, a pivotal role for IL-1 is currently emerging [4], [6], [21], [43], [44]. Notably, IL-1β was among the genes most prominently induced by live *B. burgdorferi* in PBMC (**Fig. 1AD**). Therefore, we set out to determine the contribution of IL-1 to IL-22 expression under the influence of spirochetes. For that purpose the pharmacological strategies of IL-1 receptor blockage using IL-1 receptor antagonist (IL-1Ra) and of caspase-1 inhibition were pursuit [45]. As shown in **Fig. 3A**, coincubation with IL-1Ra was able to significantly impair IL-22 secretion by PBMC indicating a role for endogenously produced IL-1 in the IL-22 response initiated by *B. burgdorferi*. This conclusion concurs with the capability of Ac-YVAD-CHO, a specific inhibitor of caspase-1, to significantly decrease IL-22 release from PBMC (**Fig. 3B**). Interestingly, IL-1β, used as single stimulus, was capable of mediating IL-22 but not IL-17 production by PBMC (**Fig. 3C**). Notably, IL-1Ra (1.3 µg/ml) did not affect IL-22 promoter activation as detected in the aforementioned Jurkat T cell protocol using conditioned media from PBMC exposed to *B. burgdorferi* as stimulus, irrespective of the presence or absence of αCD3 stimulation (data not shown). This observation is consistent with previous reports demonstrating that Jurkat T cells, in contrast to primary T cells, insufficiently respond to IL-1 due to scarce IL-1 receptor expression [46], [47]. Therefore, IL-22 promoter induction as shown in **Fig. 2DE** obviously reflects the IL-1 independent proportion of IL-22 gene activation which became evident in PBMC under the influence of IL-1Ra or Ac-YVAD-CHO (**Fig. 3AB**). As opposed to IL-1β, TNFα, used as single stimulus, was unable to stimulate secretion of IL-22 by PBMC (3.8 pg/ml±1.7 pg/ml *versus* 11.8 pg/ml±6.1 pg/ml for control *versus* TNFα (50 ng/ml), 65 h incubation, n = 5).

### T cell- and monocyte-dependent production of IL-22 by PBMC exposed to live *B. burgdorferi*

**Figure 4:**
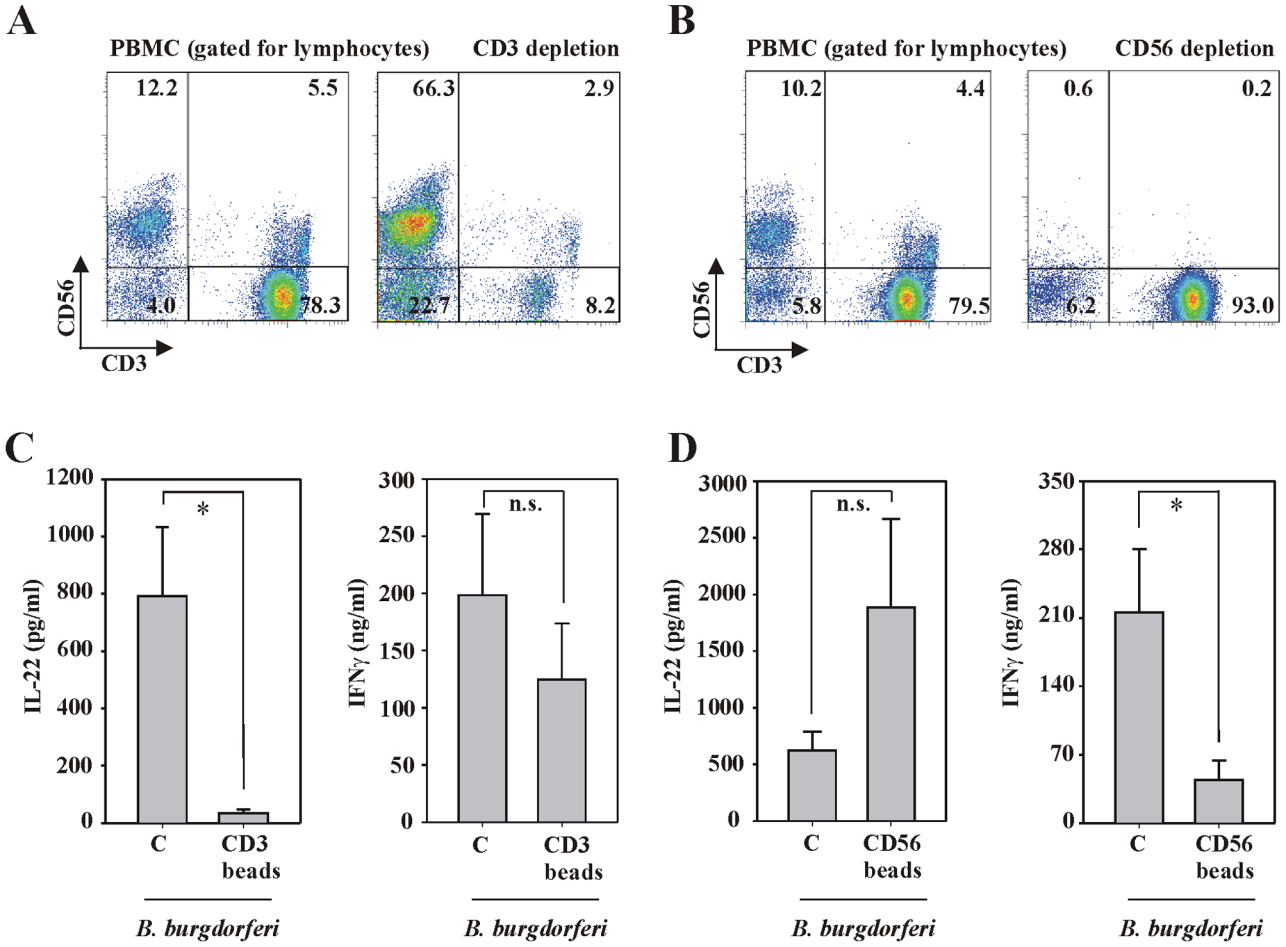
T cell-dependent production of IL-22 by PBMC exposed to live *B. burgdorferi*. After isolation, PBMC were either depleted for CD3^+^ (A) or CD56^+^ (B) cells (see methods section). FACS analysis was performed by gating on lymphocytes in order to confirm successful manipulation of PBMC. Representative depletion experiments are shown. Thereafter, whole PBMC and either CD3 (C) or CD56 (D) depleted cell populations were exposed to *B. burgdorferi* 297 (MOI = 0.1). After 65 h, release of IL-22 and IFNγ was determined by ELISA. Data are expressed as means ± SEM (CD3 depletion: n = 5; CD56 depletion: n = 8); n.s. denotes not significantly different from *B. burgdorferi* 297-stimulated whole PBMC; *p<0.05 compared with *B. burgdorferi* 297-stimulated whole PBMC. Raw data were analyzed by unpaired Student's t-test.

In order to further track down cellular sources of IL-22, subpopulations known to be capable of producing this cytokine were depleted from the mixed leukocytic population of PBMC before exposure to *B. burgdorferi*. For that purpose either cells expressing CD3 (mainly being T cells) or CD56 (mainly being NK cells) were targeted in a first set of experiments. **Fig. 4** shows representative results from CD3 (**A**) and CD56 (**B**) depletion experiments. Notably, CD3^+^ T cells were depleted by 85.2%±3.2% (n = 5) and CD56^+^ cells were depleted by 85.1%±1.8% (n = 8) from the total lymphocyte population of PBMC. In accord with previous observations [48]–[50], 4.7%±1.0% (n = 8) of lymphocytes belonged to the minor population of CD3^+^/CD56^+^ cells that includes the population of NKT cells. This CD3^+^/CD56^+^ cell population was reduced by 95.4%±1.0% (n = 8) after CD56 depletion. After the depletion procedure, equal numbers of regular PBMC or depleted cell populations were exposed to *B. burgdorferi* 297 and production of IL-22 and IFNγ was evaluated. Notably, T cell depletion (CD3^+^) from PBMC suppressed production of IL-22 in response to spirochetes down to background levels whereas IFNγ production was not significantly affected (**Fig. 4C**). In contrast, depletion of NK and NKT cells (CD56^+^) resulted in remarkable impairment of IFNγ production. This observation agrees with the previously reported dominant role of NK cells for IFNγ production by *B. burgdorferi*-stimulated PBMC [51] and furthermore emphasizes the crucial role of this cell type for initiation of inflammatory processes in the context of infections [52]. Notably, a clear tendency towards enhanced IL-22 secretion became apparent under those conditions, an observation that may in addition indicate a functional role of regulatory NK cells [53] producing modulatory factors like TGFβ [54] capable of restraining IL-1 [45] and IL-22 [26] production (**Fig. 4D**). Compared to untreated PBMC, treatment with either CD3 or CD56 microbeads (without further exposure to *Borrelia*) did not affect IL-22 or IFNγ production (data not shown).

**Figure 5:**
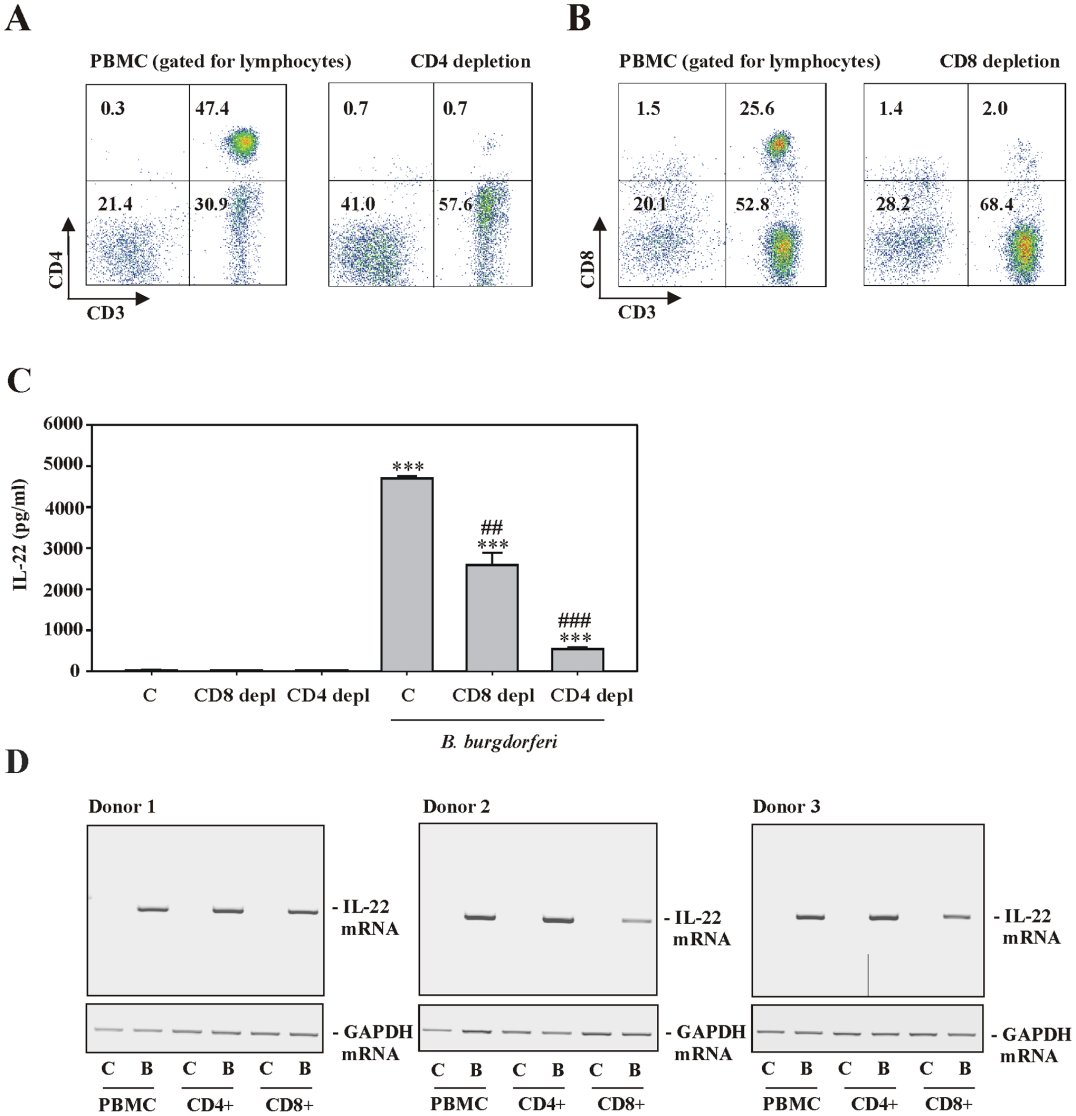
IL-22 expression by PBMC exposed to live *B. burgdorferi* is dependent on both, CD4^+^ and CD8^+^ T cells. After isolation, PBMC were either depleted for CD4^+^ (A) or CD8^+^ (B) cells (see methodssection). FACS analysis was performed by gating on lymphocytes in order to confirm successful manipulation of PBMC. Representative depletion experiments are shown. (C) Thereafter, whole PBMC and either CD4 or CD8 depleted cell populations were kept as unstimulated control or exposed to *B. burgdorferi* 297 (MOI = 0.1). After 65 h, release of IL-22 was determined by ELISA. Data are expressed as means ± SEM (n = 3); ***p<0.001 compared with unstimulated whole PBMC; ## p< 0.01, ### p<0.001 compared with whole PBMC exposed to *B. burgdorferi* 297. Raw data were analyzed by one-way ANOVA with post hoc Bonferroni correction. (D) PBMC from three different donors were either kept as unstimulated control or were exposed to *B. burgdorferi* 297 (MOI = 0.1). After 24 h, CD4^+^ and CD8^+^ T cells were isolated as outlined in the *materials and methods* section. After isolation of total RNA from either unfractionated PBMC and CD4^+^ or CD8^+^ T cells, IL-22 mRNA expression was analyzed by standard PCR. Data from all three donors are shown.

To further investigate T cell-dependent IL-22 production, CD4^+^ and CD8^+^ T cells were depleted from PBMC. **Fig. 5** shows representative results from CD4^+^ (**A**) and CD8^+^ (**B**) depletion experiments. Notably, CD4^+^ T cells were depleted by 96.9%±1.2% (n = 3) and CD8^+^ cells were depleted by 84.8%±3.7% (n = 3) from the total lymphocyte population of PBMC. After the depletion procedure, equal numbers of regular PBMC or depleted cell populations were exposed to *B. burgdorferi* 297 and production of IL-22 was evaluated. Notably, CD4^+^ or CD8^+^ T cell depletion significantly reduced production of IL-22 by 88.5% and 44.9%, respectively (**Fig. 5C**). Compared to untreated PBMC, treatment with either CD4^+^ or CD8^+^ microbeads (without further exposure to *Borrelia*) did not affect IL-22 production (**Fig. 5C**). Notably, PCR analysis of CD4^+^ and CD8^+^ T cells isolated from PBMC after exposure to *B. burgdorferi 297* revealed IL-22 expression in both T cell subsets (**Fig. 5D**). In light of the aforementioned CD3 and CD56 depletion experiments these data altogether suggest that production of IL-22 by *B. burgdorferi*-stimulated PBMC is achieved by the CD3^+^ T cell compartment.

**Figure 6:**
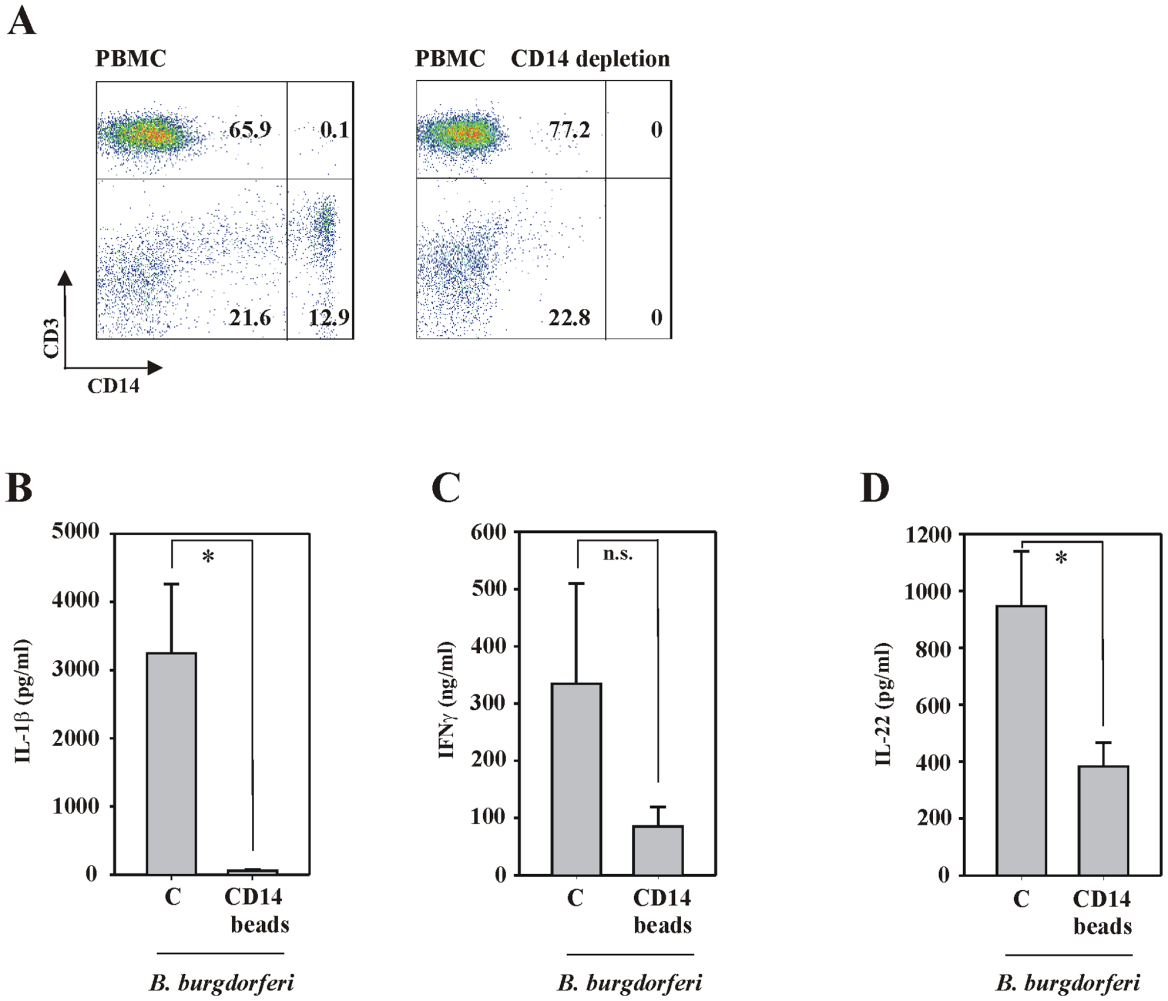
Production of IL-22 by PBMC exposed to live *B. burgdorferi* partially depends on intermediate monocyte activation. (A) After isolation, PBMC were depleted for CD14^+^ cells (see *materials and methods* section). FACS analysis was performed in order to confirm successful manipulation of PBMC. Representative depletion experiments are shown. Thereafter, whole PBMC or CD14-depleted cell populations were exposed to *B. burgdorferi* 297 (MOI = 0.1). After 65 h, release of IL-1β (B), IFNγ (C), and IL-22 (D) was determined by ELISA. Data are expressed as means ± SEM (n = 5); n.s. denotes not significantly different from *B. burgdorferi* 297-stimulated whole PBMC; *p<0.05 compared with *B. burgdorferi* 297-stimulated whole PBMC. Raw data were analyzed by unpaired Student's t-test.

Finally, we assessed whether IL-22 production by CD3^+^ T cells is dependent on the presence of CD14^+^ monocytes in *B. burgdorferi*-stimulated PBMC. **Fig. 6A** displays a representative CD14 depletion experiment. CD14^+^ cells were on average depleted by 96.8%±3.2% (n = 5) from the total population of PBMC. In fact, CD14^+^ cell depletion was associated with complete suppression of IL-1β production in the context of stimulation by *B. burgdorferi* (**Fig. 6B**), an observation in full agreement with monocytes being the major producers of IL-1β in PBMC. **Fig. 6C** also displays reduced secretion of IFNγ by the CD14^+^ depleted PBMC fraction. This tendency, likely based on weakened IL-12 production under those conditions, did however not reach statistical significance in the set of experiments performed. IL-12 in fact plays a crucial role for IFNγ production by NK cells [52]. Most notably, CD14 depletion of PBMC significantly impaired *B. burgdorferi*-mediated IL-22 production (**Fig. 6D**). Compared to untreated PBMC, treatment with CD14 microbeads (without further exposure to *Borrelia*) did not affect cytokine production (data not shown). The conclusion that IL-22 production by T cells as detected herein is dependent on activation of intermediate cell types, in particular monocytes, is furthermore emphasized by lack of significant IL-22 release from highly purified CD3^+^ T cells exposed to live *B. burgdorferi* (29.6 pg/ml±6.9 pg/ml *versus* 25.9 pg/ml±6.2 pg/ml for unstimulated CD3^+^ T cells *versus* CD3^+^ T cells exposed to *B. burgdorferi* for 65h at a MOI of 0.1; n = 6). In addition, the TLR5 ligand flagellin, a known strong activator of human PBMC [55] that is expressed by *B. burgdorferi*, activated IL-22 secretion in PBMC [56] but not in highly purified T cells (data not shown).

**Figure 7:**
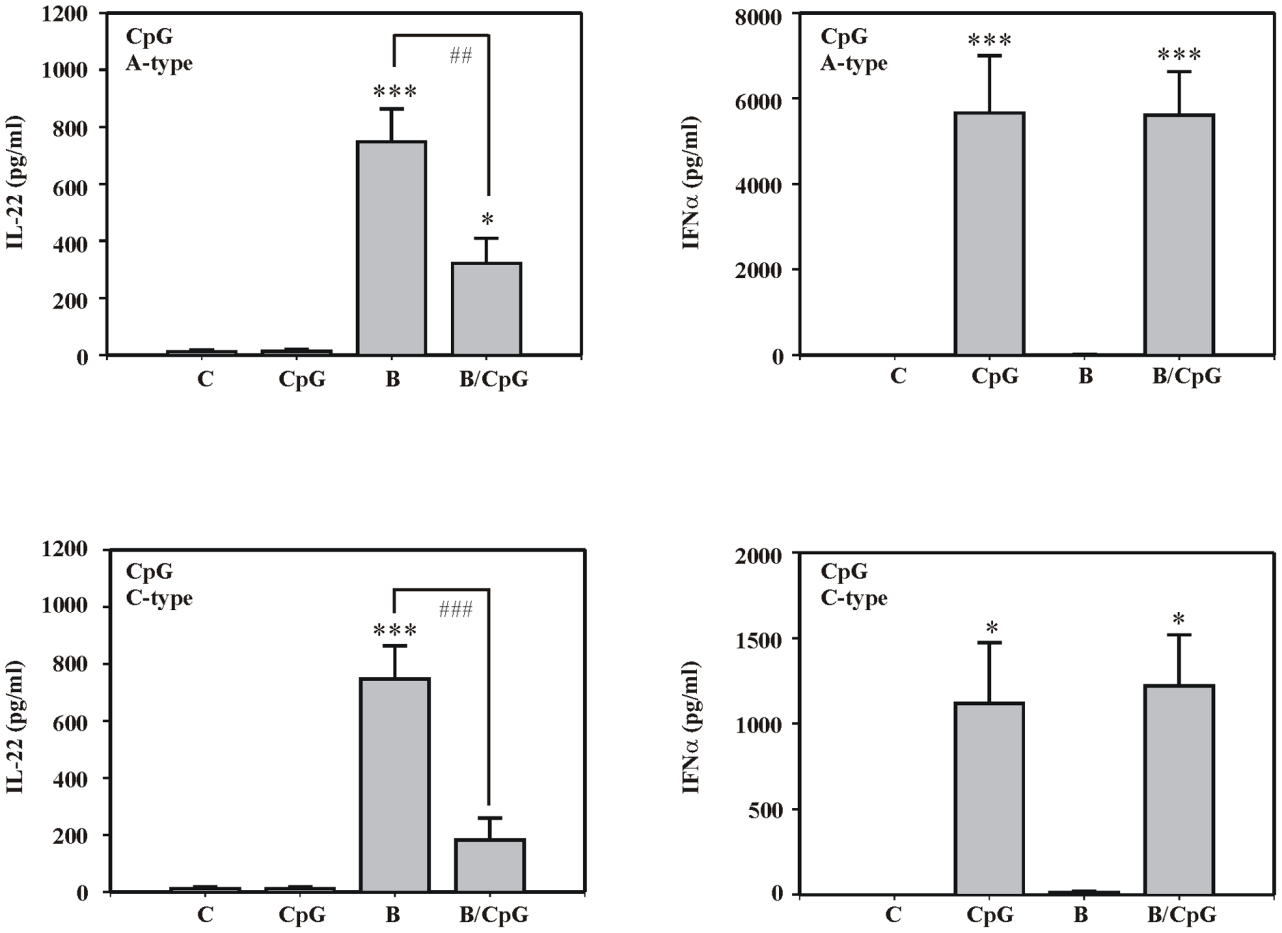
Activation of pDC by CpG oligonucleotides does not associate with enhanced production of IL-22 by PBMC. PBMC were either kept as unstimulated control, were exposed to *B. burgdorferi* 297 (MOI = 0.1) or were activated by CpG oligonucleotides (10 µg/ml) type A (A) or type C (B) alone or in the presence of *B. burgdorferi* 297 (MOI = 0.1). After 65 h, release of IL-22 (left panel) and IFNα (right panel) was determined by ELISA. Data are expressed as means ± SEM (n = 6); *p<0.05, ***p<0.001 compared with unstimulated control; ## p<0.01, ### p<0.001 compared with *B. burgdorferi* 297 alone. Raw data were analyzed by one-way ANOVA with post hoc Bonferroni correction.

A recent report, unrelated to spirochetes, demonstrates that also plasmacytoid dendritic cells (pDC) have the capability to support IL-22 production by isolated T cells activated with PMA/ionomycin [23]. To assess whether pDC may contribute to IL-22 production under the current experimental conditions, PBMC were activated by incubation with standard CpG oligonucleotides (type A and C) and production of IL-22 and IFNα was determined. Notably, pDC are the chief cell type in PBMC expressing TLR9. Engagement of the TLR9 signaling pathway by suitable CpG motifs results in efficient generation of IFNα, a common readout for pDC activation [57]. **Fig. 7** clearly demonstrates that exposure of PBMC to the CpG oligonucleotides did not mediate IL-22 production (**AB, left column**), despite induction of IFNα (**AB, right column**). Moreover, activation of the TLR9/CpG signaling axis did not enhance IL-22 release from PBMC stimulated by B. burgdorferi. Unexpectedly, CpG oligonucleotides significantly reduced IL-22 production under those conditions (**AB, left column**). As expected, *B. burgdorferi* mediated robust IL-22 production in those same experiments. At variance with a recent report [58] we did not observe production of IFNα under the influence of *B. burgdorferi* which likely reflects usage of a 100-fold lower spirochete concentration in the current study (**AB, right column)**.

### Detection of IL-22 in *erythema migrans* patients

**Figure 8:**
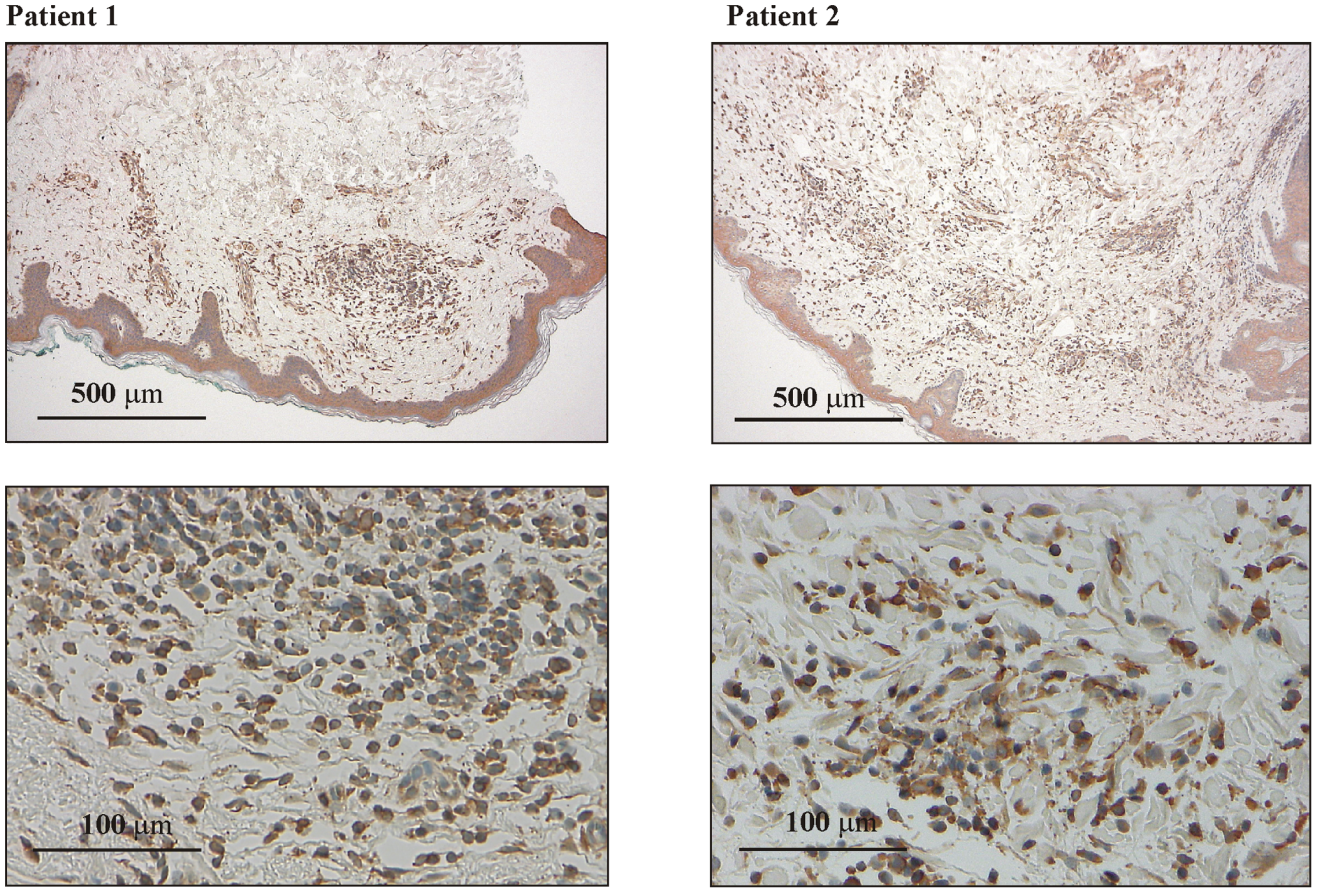
Analysis of IL-22 expression in biopsy specimens obtained from *erythema chronicum migrans* patients. 7 out of 12 specimens obtained from *erythema chronicum migrans* patients displayed abundant presence of IL-22 positive cells. Two of these skin biopsies are shown in two magnifications.

In order to relate induction of IL-22 to the pathophysiology of *erythema migrans*, immunohistochemical analysis was performed. For that purpose skin biopsies obtained from 12 patients with the diagnosis of *erythema chronicum migrans* were examined for expression of IL-22 protein. Biopsy specimens were taken a few weeks after the initial tick bite. In fact, IL-22 positive cells were detected throughout the specimens with variable degree. Abundant IL-22 was evident in 7/12 biopsy specimens. **Fig. 8** displays two selected skin biopsies from two different patients of this group. Staining was absent upon omission of the first anti-IL-22 antibody, which served as an internal control (data not shown). In the current study we were unable to provide data on IL-22 expression in normal healthy skin. However, previous observations demonstrate that IL-22 expression in normal healthy skin is barely detectably on mRNA (realtime PCR) or protein (immunohistochemistry) level, respectively [18], [59].

## Discussion

In the current study focussing on the human system the fairly unmanipulated mixed cell population of PBMC was used to analyze production of cytokines in the initial phase of leukocyte exposure to live *B. burgdorferi*. In accord with previous reports [34], a pro-inflammatory response with robust expression of key parameters such as IL-1β, TNFα, IL-12, and IFNγ was detected. However, thorough analysis likewise indicated that cytokine production not merely reflected a broad cytokine storm initiated by overwhelming innate immunity [60], [61] but actually displayed specificity. Despite being highly activated, PBMC failed to generate IL-2 and most notably IL-17, even after exposure to high concentrations of *B. burgdorferi* 297 (MOI = 30) for 65 h. Lack of immediate IL-17 production, besides IL-17A also IL-17E and IL-17F, was evident despite abundant production of IL-1β and IL-23, both regarded as pivotal factors of human Th17 development [6], [20], [21]. In contrast, activation of PBMC by IL-2/IL-23 resulted in significant IL-17 induction. Furthermore, IL-10 was not induced at a MOI of 0.1, the concentration rountinely used for experiments in the current study. Since IL-17 and IL-22 are both key to Th17-like biological activity [5], [6], we set out to analyze IL-22 under those same conditions. Surprisingly, *B. burgdorferi* mediated robust and early IL-22 expression. In fact, mRNA analysis revealed induction of IL-22 expression as early as 10 h after onset of exposure to *B. burgdorferi*. Data were complemented by immunohistochemical detection of IL-22 in skin biopsies obtained from *erythema chronicum migrans* patients, though this pathological status may barely reflect innate skin immunoactivation in the initial phase of infection.

A string of recent publications identified an apparently heterogeneous group of human T cell subsets with the capability to generate IL-22 but decisively not IL-17 [7], [23]–[27]. These cells in part belong to the memory CD4^+^ T cell subset and have been introduced as Th22 [23] or T22 cells [25]. The latter nomenclature alludes to the fact that T cells fulfilling the aforementioned criterion may display either CD4^+^ or CD8^+^ characteristics [25]. Only T cells and NK cells are regarded as relevant sources of IL-22 in human PBMC. Thus, CD3^+^/CD4^+^/CD8^+^/CD56^+^ depletion experiments suggest that CD3^+^ T cells, mainly CD4^+^ but also CD8^+^ T cells, were the principal source of PBMC-derived IL-22 under the influence of *B. burgdorferi*. Depletion experiments were complemented by PCR analysis confirming spirochete-induced IL-22 expression in CD4^+^ as well as CD8^+^ T cells. Notably, this alleged T22-type of cytokine response is achieved herein without additional artificial co-stimulatory signals such as anti-CD3/-CD28 treatment or harsh exposure to PMA/ionomycin.

Early work indicated that antigens derived from *B. burgdorferi* are in principle able to initiate activation and proliferation of human T cells [62]. However, undetectable IL-2 production by PBMC cultures even after 65 h of exposure to *B. burgdorferi* suggests that antigenic stimulation via the T cell receptor should play a lesser role in the present experimental setting.

Previous data on PBMC demonstrate that phagocytosis of *B. burgdorferi* by monocytes mediates initial activation of innate immunity with strong induction of pro-inflammatory cytokines, including IL-1β, within 4 h–8 h of exposure [32], [34], [51]. In fact, significantly increased tissue expression of IL-1β has been detected previously in biopsy specimens obtained from *erythema migrans* patients that displayed acute flu-like symptoms. Remarkably, patients of this group, particularly in the U.S.A, are more likely to exhibit hematogenous dissemination [30], possibly as a consequence of fulminant infection and subsequent collateral tissue damage. In the current cell culture model of PBMC, secretion of IL-1β is almost exclusively achieved by monocytes which, compared to macrophages, display less tight control of caspase-1 activity. Dermal tissue macrophages are obviously among the first cell types to be activated at initial infection by *B. burgdorferi*. Notably, induction and processing of pro-IL-1β by caspase-1 in dermal tissue macrophages is supposed to be achieved by a two-hit action of spirochetes delivering pathogen associated molecular patterns for activation of sensors of innate immunity along with activation of the purinoceptor P2X_7_ *via* extracellular ATP. In fact, upregulation of extracellular ATP has been observed in the context of bacterial infections and secretion of processed IL-1β [63], [64] Thus, prerequisites for caspase-1 activation and IL-1β processing/secretion in skin macrophages exposed to *B. burgdorferi* are likely in place. Since IL-1β was among the cytokines found to be most prominently upregulated by live spirochetes and this cytokine has been linked to IL-22 production in the context of Th17 activity [21], its impact on IL-22 production was assessed. In fact, blockage of IL-1 biological activity by IL-1Ra or caspase-1 inhibition impaired secretion of IL-22 by PBMC exposed to *B. burgdorferi*. CD14^+^ depletion experiments furthermore demonstrated that monocytes are responsible for practically all IL-1β being released by PBMC under the influence of *B. burgdorferi*. Moreover, depletion was associated with a significant 59.6% reduction of IL-22 release. These data indicate an important role of monocytes for T cell activation and the IL-22 response of PBMC towards live *B. burgdorferi*. On the other hand, data also suggest that about 40% of IL-22 release is monocyte- and IL-1β-independent. pDC, proven to enhance IL-22 production by isolated and PMA/ionomycin-activated T cells [23], must be regarded important in that context. However, activation of the pDC population in cultured PBMC by incubation with two different (type A and type C) CpG oligonucleotides failed to induce IL-22 herein. Those data agree with a recent publication demonstrating that systemic application of CpG oligonucleotides in mice fails to mediate IL-22 expression in the spleen and, compared to e.g. the TLR5 ligand flagellin, only moderately induces IL-22 in mesenteric lymph nodes [65]. In addition, we observed that engagement of the TLR9/CpG axis significantly impaired release of IL-22 from PBMC activated by *B. burgdorferi*. However, it should be kept in mind that exposure of pDC to CpG oligonucleotides is a cellular model but may not necessarily echo internalization of live-spirochetes by pDC. Notably, phagocytosis of *B. burgdorferi* by human DC has been detected early on [66].

Furthermore, here we report for the first time that IL-1β as sole stimulus is sufficient to mediate production of IL-22 but not of IL-17 by PBMC. This observation may have broad implications for diseases that respond to IL-1Ra treatment and have been associated with a pathologic role of IL-22. For example, it is tempting to speculate that therapeutic efficacy of IL-1Ra in some rheumatoid arthritis patients [67], [68] may in part be mediated by ill-fated IL-1-induced IL-22 in these individuals [13], [69].

Lack of early IL-17 in PBMC cultures highly activated by live *Borreliae* was unexpected. A recent report demonstrates secretion of IL-17 by naïve CD4^+^ T cells without artificial TCR activation and costimulatory signals but mediated by cocultivation with monocyte-derived Langerhans cells or DC that had been pretreated with peptidoglycan or Pam_3_CSK_4_ [70]. Both agents are known to act via the toll-like receptor (TLR)-2 and TLR2 is supposed to be one major sensor of *B. burgdorferi* [29]. Yet, previous reports clearly demonstrate that use of *B. burgdorferi* lysates and likewise more restricted TLR2 activation only poorly reflects PBMC and monocyte activation by live *B. burgdorferi* [32], [34]. Robust IL-17 production is also induced in PBMC by heat-inactivated *Candida albicans*, again in the absence of additional artificial TCR activation and costimulatory signals [71].

Quality and quantity of immediate innate immune responses is supposed to shape the course of dermal infection by *B. burgdorferi*. Specifically, expression of defined cytokine patterns may impact on bacterial dissemination. In this context, early regulation of IL-17 and IL-22 as detected herein is likely of significance. Vascular permeability is a parameter that appears to play a key role in bacterial dissemination [72]. Indeed, IL-22 has been shown to increase vascular permeability [73], a function that may relate to upregulation of matrix metalloproteinases [13], [74]–[76] and iNOS [17], [77] under the influence of this cytokine. However, despite some tendencies, there were no significant differences concerning IL-22 induction between the *Borrelia* genospecies tested, irrespective of their potential clinical properties e.g. with regard to dissemination. This observation may simply mirror the fact that IL-22 is likely only one parameter among others affecting early course of infection. On the contrary, IL-22 may actually restrain bacterial growth and dissemination by its well-characterized tissue protective and antibacterial properties [1], [2]. Certainly, this pathophysiologically important issue warrants further attention.

A crucial characteristic of IL-17 biological activity is initiation of neutrophilic inflammation [3]. Although activated neutrophils are to some degree detectable in *B. burgdorferi*-associated *erythema migrans* [31], gross infiltration by neutrophils is not a typical histopathological characteristic of these lesions. In contrast, dermal infiltrates are predominantly composed of lymphocytes and macrophages [30], [31]. Notably, neutrophil-derived products efficiently kill *B. burgdorferi*, especially when opsonized with antibodies available subsequent to initial defense [78]. Increasing dermal neutrophilic infiltration by experimental means in fact reduces bacterial dissemination in acute murine *B. burgdorferi* infection [79]. Thus, current knowledge implies that insufficient neutrophil recruitment/activation, possibly as a result of insufficient immediate IL-17 production and lack of opsonizing antibodies, may favor initial spread of *B. burgdorferi*. It must be emphasized at this point that during *B. burgdorferi*-associated chronic immunoactivation IL-17 production certainly evolves. In fact, T helper cells isolated from synovial fluid of Lyme arthritis patients produce IL-17 after polyclonal *ex vivo* stimulation [80]. Notably, blockage of IL-17 bioactivity ameliorates disease in murine Lyme arthritis [81].

The tick-*Borrelia*-skin interface shows some characteristics favoring a microenvironment that supports successful infection. Of note are diverse strategies utilized by *Borrelia* that result in impairment of host skin's innate immunity. Those include expression of CRASP proteins to impair complement-mediated killing. In addition, tick saliva displays immunosuppressive properties e.g. by its capability to decrease IL-8 and defensin secretion from human keratinocytes [29], [82]–[85]. Here we report for the first time that human PBMC exposed to live *B. burgdorferi*, despite being highly activated, lack initial IL-17 secretion. A recent report suggests that protective properties of IL-22 at host/environment interfaces are accentuated in the context of IL-17 deficiency [86]. In keeping with this line, it is tempting to speculate that IL-22 may play a decisive role in early human *B. burgdorferi* infection by supporting a balanced host defense along with tissue protection.

## Materials and Methods

### Ethics statement

For isolation of PBMC blood was taken from healthy donors. This procedure and the respective consent documents were approved by the ‘Ethik Kommission’ of the University Hospital Goethe-University Frankfurt (Geschäfts-Nr.: 170/1998). All healthy donors provided written informed consent.

### Reagents

Human recombinant IL-2, IL-23, and IL-1Ra were purchased from R&D Systems (Wiesbaden, Germany). Human IL-10 and TNFα were from TEBU-BIO/Peprotech Inc. (Frankfurt, Germany) and IL-1β was from Invitrogen/Biosource (Karlsruhe, Germany). PmxB and dexamethasone were from Calbiochem-Novabiochem GmbH (Bad Soden, Germany). Ac-YVAD-CHO was from Bachem AG (Weil am Rhein, Germany). A type A (ODN2219: ggGGGACGATCGTCgggggg) and a type C CpG oligonucleotide (ODN2395: tcgtcgttttcggcgcgcgccg) were purchased by InvivoGen (San Diego, USA).

### Bacterial isolates and culture conditions

CSF isolate *B. burgdorferi* 297, CSF isolate *B. garinii* G1, skin isolate *B. spielmanii* A14S, tick isolate *B. lusitaniae* MT-M8, CSF isolate *B. bavariensis* PBi, and clonal skin isolate *B. afzelii* FEM-1 D15 were grown at 33°C for 4 days up to cell densities of 1×10^7^ ml^−1^ in modified Barbour-Stoenner-Kelly (BSK) medium as described previously [87]. For stimulation of PBMC's, spirochetes grown to mid-log phase were harvested by centrifugation (5000× g; 30 min; 4°C) and washed twice in RPMI 1640 medium to remove bovine serum albumin and additional serum constituents of the BSK medium. The bacterial sediments were then resuspended in 500 µl of RPMI 1640 medium. Dark-field microscopy was used to check viability and motility of the spirochetes before the cells density was determined using a Kova counting chamber (Hycor Biomedical, Garden Grove, CA). In brief, an aliquot (10 µl) of the spirochetal suspension was diluted 1∶1000 or 1∶2000 in PBS. Ten microliters of the respective dilutions were drawn into the slides resulting in a homogenous distribution of the cells by capillary action. For calculation of the cell density, ten square grids were counted by using the following equation: cells/µl = average of cells per square grid×90 (multiplication factor) ×dilution as recommmend by the manufacturer.

### Serological analysis of blood donors

Sera from blood donors were tested for the presence of anti-*Borrelia* IgM and IgG antibodies using commercially available ELISAs (Enzygnost Borreliosis/IgM and Enzygnost Lyme link VlsE/IgG, Siemens Healthcare Diagnostics Products GmbH, Marburg, Germany) as well as a line immunoblot (Mikrogen, Neuried, Germany) in which p100, VlsE, p58, p41 (flagellin), p39, OspA, OspC and p18 (DbpA) are included as target antigens. For the two latter, four and five distinct OspC and p18 antigens obtained from *B. burgdorferi*, *B. afzelii*, *B. garinii*, and *B. spielmanii* were applied on the nitrocellulose membranes, respectively. Concerning the detection of anti-*Borrelia* IgM antibodies, serum samples were adsorbed using RF adsorbens prior to testing. Following the recommendations of the manufacturer for sample preparation, diluted sera were then analyzed automatically by the BEP III system. Employing line immunoassays individual serum samples were diluted 1∶50 and 1∶100 for the detection of anti-*Borrelia* IgM and anti-*Borrelia* IgG antibodies, respectively. Binding of specific antibodies was then detected by using peroxidate-conjugated goat anti-human IgM or anti-human IgG serum (1∶100).

### Isolation and cultivation of human PBMC obtained from healthy volunteers

Healthy donors had abstained from taking drugs for 2 weeks prior to the study. Blood was used from 12 donors. Eleven donors included in the study proved to be negative for IgM or IgG anti-*Borrelia* antibodies. One donor died from a traffic accident during the study and was not analyzed concerning this point. Notably, experimental results of this donor were similar to the other 11 donors. PBMC were freshly isolated from peripheral blood using Histopaque-1077 (Sigma-Aldrich, Taufkirchen, Germany) according to the manufacturer's instructions. For cultivation, PBMC were routinely resuspended in RPMI 1640 (GlutaMAX) supplemented with 10 mM HEPES and 1% human serum (Invitrogen) and seeded at 3×10^6^ cells/ml in round-bottom polypropylene tubes (Greiner, Frickenhausen, Germany). Compared to other cell culture media such as IMDM, RPMI 1640 contains a striking low amount of aromatic amino acids which may serve as precursors for ligands of the aryl hydrocarbon receptor (AhR) [88]. Notably, in the human system, AhR ligands support production of IL-22 but inhibit that of IL-17 [24]. Therefore, cultivation of human leukocytes in RPMI 1640 should technically promote IL-17 production.

### Purification of human primary CD3^+^ T cells and flow cytometric quality control

PBMC were isolated from 6 healthy donors (50 ml peripheral blood each) by density gradient centrifugation followed by T cell purification using Human T Cell Enrichment Kit (EasySep Negative Selection; StemCell Technologies, UK) and the EasySep Magnet according to the manufacturers' recommendations. Purity of viable enriched T cells and the amount of residual accessory cell populations were analyzed on a 5-color flow cytometer (FC500, Beckman Coulter, Krefeld, Germany). Monoclonal antibodies (Beckman Coulter, Marseille, France) against the following antigens (clones) were used: CD3 (UCHT1), CD4 (SFCI12T4D11), CD8 (SFCI21Thy2D3), CD14 (RMO52), CD16 (3G8), CD19 (J4.119), CD33 (D3HL60.251), CD45 (J.33), CD56 (N901), CD85k (ILT3, clone ZM3.8) and CD123 (107D2) conjugated with fluorescein isothiocyanate (FITC), phycoerythrin, (PE), phycoerythrin-Texas-Red-Tandem (ECD), phycoerythrin-cyanin-5 (PC-5) or phycoerythrin-cyanin-7 (PC-7). For assessment of viability 7-AAD was used. The panel combinations CD45/CD4/CD8/CD3/CD14 and CD45/CD56/CD19/CD3/CD16 were performed for analyzing the major leukocyte subpopulations in dual platform approach. Measurements of the myeloid dendritic cells and plasmacytoid dendritic cells were carried out in a single platform approach using fluorescent microspheres (Flow-Count fluorospheres, Beckman Coulter). T cell purification of the 6 different healthy donors resulted in a range of 99.6% to 99.9% CD3+ viable T cells (median purity: 99.8%) gated on CD45+ leukocytes. The remaining cells belonged to a median of 0.02% CD56+CD3- NK cells and less than 0.001% and 0.004% for myeloid dendritic and plasmacytoid dendritic cells, respectively. For both, CD19^+^ B cells and CD14^+^ monocytes no residual cells could be detected. After isolation, purified CD3^+^ T cells were cultured in the aforementioned PBMC medium.

### Depletion of CD3^+^, CD56^+^, CD4^+^, CD8^+^, and CD14^+^ cells from PBMC

CD3-, CD56-, CD4-, CD8-, or CD14-beads for depletion of specific cell populations were used according to the manufacturer's instructions (Miltenyi, Bergisch Gladbach, Germany). Briefly, 1.5×10^7^ PBMC were used per column (without beads as control and with specific beads) and were counted again after the depletion procedure. Cells were resuspended in aforementioned medium and seeded at 3×10^6^ cells/ml in round-bottom polypropylene tubes. To assess successful depletion, FACS analysis (either FACS Calibur or FACS Canto II, BD Biosciences, Heidelberg, Germany) was performed with the following antibodies: mouse monoclonal anti-human CD3-PerCP/Cy5.5 (Biozol, Eching, Germany), CD56-FITC (eBioscience, Frankfurt, Germany), CD4-PE-Cy7, CD8-Horizon (both BD Bioscience, Heidelberg, Germany), and CD14-PE (eBioscience, Frankfurt, Germany). FACS data were analyzed by gating on lymphocytes (CD3, CD56, CD4, CD8 depletion) or on total PBMC (CD14 depletion).

### Isolation of CD4^+^ and CD8^+^ T cells from PBMC

CD4- or CD8-beads for subset isolation were used according to the manufacturer's instructions (Miltenyi). 2.4×10^7^ PBMC were applied per column. To assess successful purification, FACS analysis (FACS Canto II) was performed with the following antibodies: mouse monoclonal anti-human CD3-PerCP/Cy5.5 (Biozol), CD4-PE-Cy7, and CD8-Horizon (BD Bioscience). FACS data were analyzed by gating on lymphocytes (purity: CD4^+^, 94.9%±2.4%; CD8^+^, 97.3%±1.0%; numbers include infrequent CD4^+^CD8^+^ T cells [89]). Subsequent isolation of total RNA and analysis for IL-22 mRNA expression by standard PCR was performed as described below.

### Cultivation of Jurkat T cells

Human Jurkat T cells were obtained from the German Collection of Microorganisms and Cell Cultures (Braunschweig, Germany). For maintainance Jurkat T cells were cultured in RPMI 1640 supplemented with 100 U/ml penicillin, 100 µg/ml streptomycin, and 10% heat-inactivated FCS (GIBCO-BRL, Eggenstein, Germany). For experiments with conditioned media Jurkat T cells were seeded on 6-well polystyrene plates (Greiner) at a density of 2.5×106 cells/ml.

### Cloning of the human IL-22 promotor, transient transfection of Jurkat T cells, and luciferase reporter assays

Using genomic DNA isolated from human THP-1 cells we amplified the 5′ flanking region of the IL-22 gene by pfu polymerase (Invitrogen). For that purpose the following primers generating a 1230 bp promotor fragment (excluding an additional flanking BglII or HindIII cloning/restriction site) were used: forward, 5′-CAATAGGTATTTGCATTTTGAT-AC-3′ and reverse, 5′-TGCAGACAATTCTAACTCGAG-3′. This promotor fragment ends 5′ adjacent to the adenine nucleotide of the IL-22 translational start site. After cloning into pGL3-Basic (Promega, Mannheim, Germany) and sequencing (Seqlab, Göttingen, Germany) this plasmid (pGL3-IL22) was transiently transfected into Jurkat T cells using DMRIE-C reagent (Invitrogen). For each reaction 4 µg of pGL3-IL22 were transfected into 2.5×10^6^ Jurkat T cells according to the manufacturer's instructions. 0.1 µg pRL-TK (Promega) coding for *Renilla* luciferase were cotransfected. After 5 h of transfection and medium change, cells were rested for 15 h. Thereafter, cells were resuspended in conditioned medium. Conditioned media for resuspension of Jurkat T cells were obtained either from unstimulated PBMC (CCM) or from PBMC exposured for 15 h to *B. burgdorferi* 297 at the indicated MOI. After resuspension, Jurkat T cells were stimulated where indicated by an mouse anti-human CD3 antibody (25 µg/ml, eBioscience). After 8 h, cells were harvested and luciferase activity was determined using the dual reporter gene system (Promega) and an automated chemiluminescence detector (Berthold, Bad Wildbad, Germany).

### Detection of IL-22 mRNA by standard and realtime PCR

Total RNA was isolated using TRI-Reagent (Sigma-Aldrich) and transcribed using random hexameric primers and Moloney virus reverse transcriptase (Applied Biosystems, Weiterstadt, Germany). The following sequences were performed for standard PCR: 94°C for 10 min (1 cycle); 94°C for 1 min, 60°C (GAPDH), 58°C (IL-22) for 30 sec, and 72°C for 45 sec (with the indicated numbers of cycles); final extension phase at 72°C for 7 min. Primer sequences and length of resulting amplicons: GAPDH (F): 5′-ACCACAGTCCATGCCA-TCAC-3′, GAPDH (R): 5′-TCCACCACCCTGTTGCTGTA-3′, 452 bp, 24 cycles; IL-22 (F): 5′-GCTAAGGAGGCTAGCTTG-3′, IL-22 (R): 5′-CAGCAAATCCAGTTCTCC-3′, 299 bp, 38 cycles. Identity of amplicons was confirmed by sequencing (310 Genetic Analyzer, Applied Biosystems). During realtime PCR, changes in fluorescence were caused by the Taq-polymerase degrading the probe that contains a fluorescent dye (FAM used for IL-22, VIC for GAPDH) and a quencher (TAMRA). For IL-22 (#Hs00220924_m1) and GAPDH (#4310884E) pre-developed assay reagents were obtained (Applied Biosystems). Assay-mix was used from Invitrogen. Realtime PCR was performed on AbiPrism 7500 Fast Sequence Detector (Applied Biosystems): One initial step at 95°C for 5 min was followed by 45 cycles at 95°C for 2 seconds and 60°C for 25 seconds. Detection of the dequenched probe, calculation of threshold cycles (Ct values), and data analysis were performed by the Sequence Detector software. Relative changes in IL-22 mRNA expression compared to unstimulated control and normalized to GAPDH were quantified by the 2^-ddCt^ method.

### Cytokine profiling by antibody array analysis

Human Cytokine Array Panel A Kit (R&D Systems) was used for analysis according to the manufacturer's instructions. PBMC were either kept as unstimulated control or exposed to live *B. burgdorferi* 297 (MOI = 0.1). After 65 h, analysis was performed by using supernatants in a 1∶3 dilution. Signals were detected by a chemoluminescence detection kit (GE Healthcare, Freiburg, Germany) according to manufacturer's instructions. Semiquantitative analysis were performed by using Quantity One software (Biorad, Munich, Germany).

### Analysis of cytokine release by emzyme-linked immunosorbent assay (ELISA)

Concentrations of IL-8, IFNγ, and TNFα, (Pharmingen/BD Biosciences), IL-1β, IL-17A (denoted IL-17 throughout the manuscript), IL-22, IL-23, and IL-18 (R&D Systems/MBL), IFNα (BenderMed Systems, Vienna, Austria), as well as IL-2, IL-10, IL-17F, and IL12p70 (eBioscience) in cell-free cell culture supernatants were determined by ELISA according to the manufacturers' instructions.

### Immunohistochemical detection of IL-22 in tissue sections obtained from *erythema chronicum migrans* patients

Ready-to-use tissue sections (Dermatopathological Laboratory Offenbach) obtained from lesions of patients diagnosed for *erythema chronicum migrans* after a tick bite were immunohistochemically analyzed. Biopsy specimens were taken a few weeks after the initial tick bite. Four-micrometer sections of formalin-fixed, paraffin-embedded skin lesion from *erythema migrans* patients were used for detection of cutaneous cells producing IL-22. Briefly, sections were deparaffinized and unmasked by heat treatment (Dako Cytomation Target Retrieval Solution). Thereafter, sections were incubated with rabbit anti-human IL-22 polyclonal antibody (Abcam, Cambridge, UK) overnight at 4°C. Biotinylated-goat anti-rabbit antibody and the avidin-biotin-peroxidase complex (ABC-system, Santa Cruz) and 3,3-diaminobenzidine-tetra-hydrochloride pellets (Sigma-Aldrich) were used for detection.

### Statistical analysis

Data are shown as means ± SEM (PBMC) or means ± SD (Jurkat T cells) and are presented as pg/ml, ng/ml, fold-induction, (% of αCD3 at CCM), (% of CCM), or (% of *B. burgdorferi* alone). Statistical analysis was performed either by unpaired Students *t*-test or one-way ANOVA with post-hoc Bonferroni correction as indicated in the legends (GraphPad 5.0).

## Supporting Information

Figure S1 *B. burgdorferi* 297-induced cytokine expression as detected by antibody array analysis. Antibody array overview.
(0.04 MB DOC)

Figure S2 *B. burgdorferi* 297-induced cytokine expression as detected by antibody array analysis. Densitometric analysis of Fig. 1A.
(0.29 MB TIF)

## References

[1] Aujla SJ, Kolls JK (2009). IL-22: a critical mediator in mucosal host defense.. J Mol Med.

[2] Wolk K, Witte E, Witte K, Warszawska K, Sabat R (2010). Biology of interleukin-22.. Semin Immunopathol.

[3] Gaffen SL (2008). An overview of IL-17 function and signaling.. Cytokine.

[4] Miossec P, Korn T, Kuchroo VK (2009). Interleukin-17 and type 17 helper T cells.. N Engl J Med.

[5] Steinman L (2007). A brief history of T(H)17, the first major revision in the T(H)1/T(H)2 hypothesis of T cell-mediated tissue damage.. Nat Med.

[6] Louten J, Boniface K, de Waal Malefyt R (2009). Development and function of TH17 cells in health and disease.. J Allergy Clin Immunol.

[7] Yssel H, Pène J (2009). Interleukin-22-producing T cells: a specialized population involved in skin inflammation?. Immunol Cell Biol.

[8] Colonna M (2009). Interleukin-22-producing natural killer cells and lymphoid tissue inducer-like cells in mucosal immunity.. Immunity.

[9] Zheng Y, Valdez PA, Danilenko DM, Hu Y, Sa SM (2008). Interleukin-22 mediates early host defense against attaching and effacing bacterial pathogens.. Nat Med.

[10] Aujla SJ, Chan YR, Zheng M, Fei M, Askew DJ (2008). IL-22 mediates mucosal host defense against Gram-negative bacterial pneumonia.. Nat Med.

[11] Ziesché E, Scheiermann P, Bachmann M, Sadik CD, Hofstetter C (2009). Dexamethasone suppresses interleukin-22 associated with bacterial infection in vitro and in vivo.. Clin Exp Immunol.

[12] Bingold TM, Ziesché E, Scheller B, Sadik CD, Franck K (2010). Interleukin-22 detected in patients with abdominal sepsis.. Shock.

[13] Geboes L, Dumoutier L, Kelchtermans H, Schurgers E, Mitera T (2009). Proinflammatory role of the Th17 cytokine interleukin-22 in collagen-induced arthritis in C57BL/6 mice.. Arthritis Rheum.

[14] Zheng Y, Danilenko DM, Valdez P, Kasman I, Eastham-Anderson J (2007). Interleukin-22, a T(H)17 cytokine, mediates IL-23-induced dermal inflammation and acanthosis.. Nature.

[15] Sugimoto K, Ogawa A, Mizoguchi E, Shimomura Y, Andoh A (2008). IL-22 ameliorates intestinal inflammation in a mouse model of ulcerative colitis.. J Clin Invest.

[16] Zenewicz LA, Yancopoulos GD, Valenzuela DM, Murphy AJ, Stevens S (2008). Innate and adaptive interleukin-22 protects mice from inflammatory bowel disease.. Immunity.

[17] Ziesché E, Bachmann M, Kleinert H, Pfeilschifter J, Mühl H (2007). The interleukin-22/STAT3 pathway potentiates expression of inducible nitric-oxide synthase in human colon carcinoma cells.. J Biol Chem.

[18] Boniface K, Guignouard E, Pedretti N, Garcia M, Delwail A (2007). A role for T cell-derived interleukin 22 in psoriatic skin inflammation.. Clin Exp Immunol.

[19] Ma HL, Liang S, Li J, Napierata L, Brown T (2008). IL-22 is required for Th17 cell-mediated pathology in a mouse model of psoriasis-like skin inflammation.. J Clin Invest.

[20] Annunziato F, Romagnani S (2009). Do studies in humans better depict Th17 cells?. Blood.

[21] Wilson NJ, Boniface K, Chan JR, McKenzie BS, Blumenschein BM (2007). Development, cytokine profile and function of human interleukin 17-producing helper T cells.. Nat Immunol.

[22] Amadi-Obi A, Yu CR, Liu X, Mahdi RM, Clarke GL (2007). TH17 cells contribute to uveitis and scleritis and are expanded by IL-2 and inhibited by IL-27/STAT1.. Nat Med.

[23] Duhen T, Geiger R, Jarrossay D, Lanzavecchia A, Sallusto F (2009). Production of interleukin 22 but not interleukin 17 by a subset of human skin-homing memory T cells.. Nat Immunol.

[24] Trifari S, Kaplan CD, Tran EH, Crellin NK, Spits H (2009). Identification of a human helper T cell population that has abundant production of interleukin 22 and is distinct from T(H)-17, T(H)1 and T(H)2 cells.. Nat Immunol.

[25] Nograles KE, Zaba LC, Shemer A, Fuentes-Duculan J, Cardinale I (2009). IL-22-producing “T22” T cells account for upregulated IL-22 in atopic dermatitis despite reduced IL-17-producing TH17 T cells.. J Allergy Clin Immunol.

[26] Volpe E, Touzot M, Servant N, Marloie-Provost MA, Hupe P (2009). Multiparametric analysis of cytokine-driven human Th17 differentiation reveals a differential regulation of IL-17 and IL-22 production.. Blood.

[27] Eyerich S, Eyerich K, Pennino D, Carbone T, Nasorri F (2009). Th22 cells represent a distinct human T cell subset involved in epidermal immunity and remodeling.. J Clin Invest.

[28] Hengge UR, Tannapfel A, Tyring SK, Erbel R, Arendt G, Ruzicka T (2003). Lyme borreliosis.. Lancet Infect Dis.

[29] Rupprecht TA, Koedel U, Fingerle V, Pfister HW (2008). The pathogenesis of lyme neuroborreliosis: from infection to inflammation.. Mol Med.

[30] Müllegger RR, McHugh G, Ruthazer R, Binder B, Kerl H (2000). Differential expression of cytokine mRNA in skin specimens from patients with erythema migrans or acrodermatitis chronica atrophicans.. J Invest Dermatol.

[31] Salazar JC, Pope CD, Sellati TJ, Feder HM, Kiely TG (2003). Coevolution of markers of innate and adaptive immunity in skin and peripheral blood of patients with erythema migrans.. J Immunol.

[32] Miller LC, Isa S, Vannier E, Georgilis K, Steere AC (1992). Live Borrelia burgdorferi preferentially activate interleukin-1 beta gene expression and protein synthesis over the interleukin-1 receptor antagonist.. J Clin Invest.

[33] Cruz AR, Moore MW, La Vake CJ, Eggers CH, Salazar JC (2008). Phagocytosis of Borrelia burgdorferi, the Lyme disease spirochete, potentiates innate immune activation and induces apoptosis in human monocytes.. Infect Immun.

[34] Salazar JC, Duhnam-Ems S, La Vake C, Cruz AR, Moore MW (2009). Activation of human monocytes by live Borrelia burgdorferi generates TLR2-dependent and -independent responses which include induction of IFN-beta.. PLoS Pathog.

[35] Härtel C, Bein G, Müller-Steinhardt M, Klüter H (2001). Ex vivo induction of cytokine mRNA expression in human blood samples.. J Immunol Methods.

[36] Porat R, Poutsiaka DD, Miller LC, Granowitz EV, Dinarello CA (1992). Interleukin-1 (IL-1) receptor blockade reduces endotoxin and Borrelia burgdorferi-stimulated IL-8 synthesis in human mononuclear cells.. FASEB J.

[37] Sadik CD, Hunfeld KP, Bachmann M, Kraiczy P, Eberhardt W (2008). Systematic analysis highlights the key role of TLR2/NF-kappaB/MAP kinase signaling for IL-8 induction by macrophage-like THP-1 cells under influence of Borrelia burgdorferi lysates.. Int J Biochem Cell Biol.

[38] Poutsiaka DD, Clark BD, Vannier E, Dinarello CA (1991). Production of interleukin-1 receptor antagonist and interleukin-1 beta by peripheral blood mononuclear cells is differentially regulated.. Blood.

[39] Kaliora AC, Stathopoulou MG, Triantafillidis JK, Dedoussis GV, Andrikopoulos NK (2007). Alterations in the function of circulating mononuclear cells derived from patients with Crohn's disease treated with mastic.. World J Gastroenterol.

[40] Lun SW, Wong CK, Ko FW, Ip WK, Hui DS (2006). Aberrant expression of CC and CXC chemokines and their receptors in patients with asthma.. J Clin Immunol.

[41] Scriba TJ, Kalsdorf B, Abrahams DA, Isaacs F, Hofmeister J (2008). Distinct, specific IL-17- and IL-22-producing CD4+ T cell subsets contribute to the human anti-mycobacterial immune response.. J Immunol.

[42] Mühl H, Pfeilschifter J (2003). Anti-inflammatory properties of pro-inflammatory interferon-gamma.. Int Immunopharmacol.

[43] Chen Z, Tato CM, Muul L, Laurence A, O'Shea JJ (2007). Distinct regulation of interleukin-17 in human T helper lymphocytes.. Arthritis Rheum.

[44] Acosta-Rodriguez EV, Napolitani G, Lanzavecchia A, Sallusto F (2007). Interleukins 1beta and 6 but not transforming growth factor-beta are essential for the differentiation of interleukin 17-producing human T helper cells.. Nat Immunol.

[45] Dinarello CA (1996). Biologic basis for interleukin-1 in disease.. Blood.

[46] Heguy A, Baldari CT, Censini S, Ghiara P, Telford JL (1993). A chimeric type II/type I interleukin-1 receptor can mediate interleukin-1 induction of gene expression in T cells.. J Biol Chem.

[47] Kuno K, Okamoto S, Hirose K, Murakami S, Matsushima K (1993). Structure and function of the intracellular portion of the mouse interleukin 1 receptor (type I). Determining the essential region for transducing signals to activate the interleukin 8 gene.. J Biol Chem.

[48] Dunne J, Lynch S, ÒFarrelly C, Todryk S, Hegarty JE (2001). Selective expansion and partial activation of human NK cells and NK receptor-positive T cells by IL-2 and IL-15.. J Immunol.

[49] Koreck A, Suranyi A, Szöny BJ, Farkas A, Bata-Csörgö Z (2002). CD3+CD56+ NK T cells are significantly decreased in the peripheral blood of patients with psoriasis.. Clin Exp Immunol.

[50] Saikh KU, Dyas B, Kissner T, Ulrich RG (2003). CD56+-T-cell responses to bacterial superantigens and immune recognition of attenuated vaccines.. Clin Diagn Lab Immunol.

[51] Moore MW, Cruz AR, LaVake CJ, Marzo AL, Eggers CH (2007). Phagocytosis of Borrelia burgdorferi and Treponema pallidum potentiates innate immune activation and induces gamma interferon production.. Infect Immun.

[52] Copper MA, Colonna M, Yokoyoma WM (2009). Hidden talents of natural killers: NK cells in innate and adaptive immunity.. EMBO Rep.

[53] Vivier E, Ugolini S (2009). Regulatory natural killer cells: new players in the IL-10 anti-inflammatory response.. Cell Host Microbe.

[54] Horwitz DA, Gray JD, Ohtsuka K, Hirokawa M, Takahashi T (1997). The immunoregulatory effects of NK cells: the role of TGF-beta and implications for autoimmunity.. Immunol Today.

[55] Bachmann M, Horn K, Poleganov MA, Paulukat J, Nold M (2006). Interleukin-18 secretion and Th1-like cytokine responses in human peripheral blood mononuclear cells under the influence of the toll-like receptor-5 ligand flagellin.. Cell Microbiol.

[56] Ziesché E (2008). Untersuchungen zur Bildung und Funktion von IL-22 bei entzündungsbedingter Immunaktivierung..

[57] Hornung V, Rothenfusser S, Britsch S, Krug A, Jahrsdörfer B (2002). Quantitative expression of toll-like receptor 1-10 mRNA in cellular subsets of human peripheral blood mononuclear cells and sensitivity to CpG oligodeoxynucleotides.. J Immunol.

[58] Petzke MM, Brooks A, Krupna MA, Mordue D, Schwartz I (2009). Recognition of Borrelia burgdorferi, the Lyme disease spirochete, by TLR7 and TLR9 induces a type I IFN response by human immune cells.. J Immunol.

[59] Guttman-Yassky E, Lowes MA, Fuentes-Duculan J, Zaba LC, Cardinale I (2008). Low expression of the IL-23/Th17 pathway in atopic dermatitis compared to psoriasis.. J Immunol.

[60] Singh SK, Girschick HJ (2006). Toll-like receptors in Borrelia burgdorferi-induced inflammation.. Clin Microbiol Infect.

[61] Schröder NW, Eckert J, Stübs G, Schumann RR (2008). Immune responses induced by spirochetal outer membrane lipoproteins and glycolipids.. Immunobiology.

[62] Roessner K, Fikrig E, Russell JQ, Cooper SM, Flavell RA (1994). Prominent T lymphocyte response to Borrelia burgdorferi from peripheral blood of unexposed donors.. Eur J Immunol.

[63] Netea MG, Simon A, van de Veerdonk F, Kullberg BJ, Van der Meer JW (2010). IL-1beta processing in host defense: beyond the inflammasomes.. PLoS Pathog.

[64] Ferrari D, Pizzirani C, Adinolfi E, Lemoli RM, Curti A (2006). The P2X7 receptor: a key player in IL-1 processing and release.. J Immunol.

[65] Van Maele L, Carnoy C, Cayet D, Songhet P, Dumoutier L (2010). TLR5 signaling stimulates the innate production of IL-17 and IL-22 by CD3(neg)CD127+ immune cells in spleen and mucosa.. J Immunol.

[66] Filgueira L, Nestlé FO, Rittig M, Joller HI, Groscurth P (1996). Human dendritic cells phagocytose and process Borrelia burgdorferi.. J Immunol.

[67] Cunnane G, Madigan A, Murphy E, FitzGerald O, Bresnihan B (2001). The effects of treatment with interleukin-1 receptor antagonist on the inflamed synovial membrane in rheumatoid arthritis.. Rheumatology (Oxford).

[68] Kalliolias GD, Liossis SN (2008). The future of the IL-1 receptor antagonist anakinra: from rheumatoid arthritis to adult-onset Still's disease and systemic-onset juvenile idiopathic arthritis.. Expert Opin Investig Drugs.

[69] Ikeuchi H, Kuroiwa T, Hiramatsu N, Kaneko Y, Hiromura K (2005). Expression of interleukin-22 in rheumatoid arthritis: potential role as a proinflammatory cytokine.. Arthritis Rheum.

[70] Aliahmadi E, Gramlich R, Grützkau A, Hitzler M, Krüger M (2009). TLR2-activated human langerhans cells promote Th17 polarization via IL-1beta, TGF-beta and IL-23.. Eur J Immunol.

[71] Zhou M, Yang B, Ma R, Wu C (2008). Memory Th-17 cells specific for C. albicans are persistent in human peripheral blood.. Immunol Lett.

[72] Szczepanski A, Benach JL (1991). Lyme borreliosis: host responses to Borrelia burgdorferi.. Microbiol Rev.

[73] Kebir H, Kreymborg K, Ifergan I, Dodelet-Devillers A, Cayrol R (2007). Human TH17 lymphocytes promote blood-brain barrier disruption and central nervous system inflammation.. Nat Med.

[74] Mikami Y, Dobschütz EV, Sommer O, Wellner U, Unno M (2009). Matrix metalloproteinase-9 derived from polymorphonuclear neutrophils increases gut barrier dysfunction and bacterial translocation in rat severe acute pancreatitis.. Surgery.

[75] Zhao Z, Chang H, Trevino RP, Whren K, Bhawan J (2003). Selective up-regulation of matrix metalloproteinase-9 expression in human erythema migrans skin lesions of acute lyme disease.. J Infect Dis.

[76] Andoh A, Zhang Z, Inatomi O, Fujino S, Deguchi Y (2005). Interleukin-22, a member of the IL-10 subfamily, induces inflammatory responses in colonic subepithelial myofibroblasts.. Gastroenterology.

[77] Iuvone T, D'Acquisto F, Van Osselaer N, Di Rosa M, Carnuccio R (1998). Evidence that inducible nitric oxide synthase is involved in LPS-induced plasma leakage in rat skin through the activation of nuclear factor-kappaB.. Br J Pharmacol.

[78] Lusitani D, Malawista SE, Montgomery RR (2002). Borrelia burgdorferi are susceptible to killing by a variety of human polymorphonuclear leukocyte components.. J Infect Dis.

[79] Xu Q, Seemanapalli SV, Reif KE, Brown CR, Liang FT (2007). Increasing the recruitment of neutrophils to the site of infection dramatically attenuates Borrelia burgdorferi infectivity.. J Immunol.

[80] Infante-Duarte C, Horton HF, Byrne MC, Kamradt T (2000). Microbial lipopeptides induce the production of IL-17 in Th cells.. J Immunol.

[81] Burchill MA, Nardelli DT, England DM, DeCoster DJ, Christopherson JA (2003). Inhibition of interleukin-17 prevents the development of arthritis in vaccinated mice challenged with Borrelia burgdorferi.. Infect Immun.

[82] Kraiczy P, Skerka C, Kirschfink M, Zipfel PF, Brade V (2002). Immune evasion of Borrelia burgdorferi: insufficient killing of the pathogens by complement and antibody.. Int J Med Microbiol.

[83] Kraiczy P, Hellwage J, Skerka C, Becker H, Kirschfink M (2004). Complement resistance of Borrelia burgdorferi correlates with the expression of BbCRASP-1, a novel linear plasmid-encoded surface protein that interacts with human factor H and FHL-1 and is unrelated to Erp proteins.. J Biol Chem.

[84] Hovius JW (2009). Spitting image: tick saliva assists the causative agent of Lyme disease in evading host skin's innate immune response.. J Invest Dermatol.

[85] Marchal CM, Luft BJ, Yang X, Sibilia J, Jaulhac B (2009). Defensin is suppressed by tick salivary gland extract during the in vitro interaction of resident skin cells with Borrelia burgdorferi.. J Invest Dermatol.

[86] Sonnenberg GF, Nair MG, Kirn KJ, Zaph C, Fouser LA (2010). Pathological versus protective functions of IL-22 in airway inflammation are regulated by IL-17A.. J Exp Med.

[87] Kraiczy P, Skerka C, Brade V, Zipfel PF (2001). Further characterization of complement regulator-acquiring surface proteins of *Borrelia burgdorferi*.. Infect Immun.

[88] Veldhoen M, Hirota K, Christensen J, O'Garra A, Stockinger B (2009). Natural agonists for aryl hydrocarbon receptor in culture medium are essential for optimal differentiation of Th17 T cells.. J Exp Med.

[89] Nascimbeni M, Shin EC, Chiriboga L, Kleiner DE, Rehermann B (2004). Peripheral CD4(+)CD8(+) T cells are differentiated effector memory cells with antiviral functions.. Blood.

