# Supplementary material for: Early Production of IL-22 but Not IL-17 by Peripheral Blood Mononuclear Cells Exposed to live Borrelia burgdorferi: The Role of Monocytes and Interleukin-1
Source: PLoS Pathog. 2010 Oct 14;6(10):e1001144. doi: 10.1371/journal.ppat.1001144 (PMC2954834; doi:10.1371/journal.ppat.1001144)
Supplement: Figure S1 — B. burgdorferi 297-induced cytokine expression as detected by antibody array analysis. Antibody array overview. (0.04 MB DOC) [file ppat.1001144.s001.doc]

**Supplemental Figure 1: Overview on the antibody array (see Figure 1A)**

| **Pos** | **Pos** | **C5a** | **C5a** | **CD154** | **CD154** | **G-CSF** | **G-CSF** | **GM-CSF** | **GM-CSF** | **GRO α** | **GRO α** | **I-309** | **I-309** | **sICAM-1** | **sICAM-1** | **IFN-γ** | **IFN- γ** | **Pos** | **Pos** |
| --- | --- | --- | --- | --- | --- | --- | --- | --- | --- | --- | --- | --- | --- | --- | --- | --- | --- | --- | --- |
|  |  | **IL-1 α** | **IL-1 α** | **IL-1β** | **IL-1 β** | **IL-1Ra** | **IL-1Ra** | **IL-2** | **IL-2** | **IL-4** | **IL-4** | **IL-5** | **IL-5** | **IL-6** | **IL-6** | **IL-8** | **IL-8** |  |  |
|  |  | **IL-10** | **IL-10** | **IL-12p70** | **IL-12p70** | **IL-13** | **IL-13** | **IL-16** | **IL-16** | **IL-17** | **IL-17** | **IL-17E** | **IL-17E** | **IL-23** | **IL-23** | **IL-27** | **IL-27** |  |  |
|  |  | **IL-32α** | **IL-32 α** | **IP-10** | **IP-10** | **I-TAC** | **I-TAC** | **MCP-1** | **MCP-1** | **MIF** | **MIF** | **MIP-1 α** | **MIP-1 α** | **MIP-1 β** | **MIP-1 β** | **PAI-1** | **PAI-1** |  |  |
| **Pos** | **Pos** | **RANTES** | **RANTES** | **SDF-1** | **SDF-1** | **TNF- α** | **TNF- α** | **sTREM-1** | **sTREM-1** |  |  |  |  |  |  |  |  | **Neg** | **Neg** |
